# Effectiveness and patient safety of platelet aggregation inhibitors in the prevention of cardiovascular disease and ischemic stroke in older adults – a systematic review

**DOI:** 10.1186/s12877-017-0572-7

**Published:** 2017-10-16

**Authors:** Maren Meinshausen, Anja Rieckert, Anna Renom-Guiteras, Moritz Kröger, Christina Sommerauer, Ilkka Kunnamo, Yolanda V. Martinez, Aneez Esmail, Andreas Sönnichsen

**Affiliations:** 1 Institute of General Practice and Family Medicine, Faculty of Health, University of Witten/Herdecke, Alfred-Herrhausen-Straße, Witten, Germany; 2 Department of Geriatrics, In the University Hospital Parc de Salut Mar, Passeig Marítim, Barcelona, Spain; 3 Duodecim Medical Publications Ltd, Kalevankatu, Helsinki, Finland; 4 NIHR School for Primary Care Research, Manchester Academic Health Science Centre, Oxford Rd, Manchester, UK; 5 NIHR Greater Manchester Patient Safety Translational Research Centre, Manchester Academic Health Science Centre, Manchester, UK

**Keywords:** Antiplatelet drugs, Platelet aggregation inhibitors, Polypharmacy, Antiplatelet therapy, Acetylsalicylic acid, Clopidogrel, Primary prevention, Secondary prevention, Cardiovascular disease, Cerebrovascular disease, Peripheral artery occlusive disease, Aged, Deprescribing

## Abstract

**Background:**

Platelet aggregation inhibitors (PAI) are among the most frequently prescribed drugs in older people, though evidence about risks and benefits of their use in older adults is scarce. The objectives of this systematic review are firstly to identify the risks and benefits of their use in the prevention and treatment of vascular events in older adults, and secondly to develop recommendations on discontinuing PAI in this population if risks outweigh benefits.

**Methods:**

Staged systematic review consisting of three searches. Searches 1 and 2 identified systematic reviews and meta-analyses. Search 3 included controlled intervention and observational studies from review-articles not included in searches 1 and 2. All articles were assessed by two independent reviewers regarding the type of study, age of participants, type of intervention, and clinically relevant outcomes. After data extraction and quality appraisal we developed recommendations to stop the prescribing of specific drugs in older adults following the Grading of Recommendations Assessment Development and Evaluation (GRADE) methodology.

**Results:**

Overall, 2385 records were screened leading to an inclusion of 35 articles reporting on 22 systematic reviews and meta-analyses, 11 randomised controlled trials, and two observational studies. Mean ages ranged from 57.0 to 84.6 years. Ten studies included a subgroup analysis by age. Overall, based on the evaluated evidence, three recommendations were formulated. First, the use of acetylsalicylic acid (ASA) for primary prevention of cardiovascular disease (CVD) in older people cannot be recommended due to an uncertainty in the risk-benefit ratio (weak recommendation; low quality of evidence). Secondly, the combination of ASA and clopidogrel in patients without specific indications should be avoided (strong recommendation; moderate quality of evidence). Lastly, to improve the effectiveness and reduce the risks of stroke prevention therapy in older people with atrial fibrillation (AF) and a CHA_2_DS_2_-VASc score of ≥ 2, the use of ASA for the primary prevention of stroke should be discontinued in preference for the use of oral anticoagulants (weak recommendation; low quality of evidence).

**Conclusions:**

The use of ASA for the primary prevention of CVD and the combination therapy of ASA and clopidogrel for the secondary prevention of vascular events in older people may not be justified. The use of oral anticoagulants instead of ASA in older people with atrial fibrillation may be recommended. Further high quality studies with older adults are needed.

**Electronic supplementary material:**

The online version of this article (doi:10.1186/s12877-017-0572-7) contains supplementary material, which is available to authorized users.

## Background

There is evidence that the use of multiple medications has been rising over the past years, especially among older people [1]. Platelet aggregation inhibitors (PAI) constitute some of the most frequently prescribed drugs among people aged ≥65 [2, 3]. They are indicated in the prevention of cardiovascular disease, during and after myocardial infarction or acute coronary syndrome, during and after angioplasty and stenting, in the prevention of stroke and transient ischaemic attacks (TIA), and in the prevention of peripheral artery occlusive disease [4–6]. The pharmacological mechanism of action of PAI is the inhibition of thrombocyte activation and/or impeding aggregation. The treatment goal is preventing thrombotic complications [7]. However, an undesirable effect of this platelet inhibition is an increase in the risk of bleeding [8].

Despite the benefit of reducing cardiovascular events, several studies show that PAI are frequently associated with hospital admission due to adverse drug events [9–12]. Some of these adverse drug events could be avoided, for instance by an increased monitoring of the use of drugs and regular medication reviews [9–11]. In the case of acetylsalicylic acid (ASA), secondary prophylaxis with Helicobacter pylori eradication and proton pump inhibitors reduces the risk of gastrointestinal bleeding [9].

The use of PAI has been questioned in older people due to a higher risk of adverse events compared to younger, healthier adults [13, 14]. This higher risk is attributable to changes in pharmacokinetics and pharmacodynamics and a higher risk of drug interactions in older people [15].

Evidence regarding the risks and benefits of antiplatelet drugs in older people is scarce, as most studies focus on younger patients with fewer co-morbidities [16]. Existing guidelines usually do not adapt for old age and multimorbidity [17]. Hence, the balance between risks and benefits of PAI in the management of cerebrovascular disease, peripheral artery occlusive disease, and coronary disease in older adults with multimorbidity is not clear [12]. We therefore set out to systematically review the available evidence regarding the use of PAI in older and multimorbid people.

The objectives of this Systematic Review (SR) are

- To identify the risks and benefits of the use of PAI in the treatment or prevention of cerebrovascular disease, peripheral artery occlusive disease, and coronary disease in older adults.
- To develop recommendations which will enable physicians to stop the use of PAI in the treatment or prevention of cerebrovascular disease, peripheral artery occlusive disease, and coronary disease in older adults based on current best evidence.

The developed stop-recommendations will be incorporated in an electronic decision support tool for general practitioners within the EU-Project PRIMA-eDS (Polypharmacy in chronic diseases: Reduction of Inappropriate Medication and Adverse drug events in older populations by electronic Decision Support) [18].

## Methods

A SR was performed in accordance with the methodology described earlier [19] following a specific study protocol (available from the authors upon request). We will report on the results narratively.

### Search strategy

As described in the publication of our methodology [19], we employed a step-wise approach that consisted of four searches, of which the consecutive one was only conducted when the prior one did not lead to recent and high quality results. Search 1 was targeted at SR and meta-analyses (MA) in the Cochrane Database of Systematic Reviews (OVID interface, 2005 onwards) and Database of Abstracts or Reviews of Effects (DARE, OVID interface, 1991 onwards). Search 2 was also directed at SR and MA, but extended to MEDLINE (OVID interface, 1946 onwards), EMBASE (OVID interface, 1974 onwards), Health Technology Assessment (HTA, OVID interface 2001 onwards), and International Pharmaceutical Abstracts (IPA, OVID interface 1970 onwards). Search 3a was performed to find single studies (randomized controlled trials (RCT) and observational studies (OS) from SR and MA not included in searches 1 and 2 due to not meeting our inclusion criteria but containing eligible studies. Search 3b looked for RCT and OS in MEDLINE, EMBASE, HTA and IPA.

For this SR, searches 1 and 2 were performed in December 8th, 2015. The search string (see additional file 1) was developed with the help of a PICOS (population, intervention, comparison, outcomes and study design) framework. In search 3a, we identified eligible randomised controlled trials and OS from SR and MA, which themselves were not eligible for inclusion in our review (mainly because they were not focussed at older people). In parallel to the study selection of searches 1 and 2, we prepared a list of references to be checked in search 3a. Search 3b was considered as not being necessary because the SR and MA retrieved covered all eligible studies (see results) and we did not expect to find any additional eligible studies. In addition to database searches, all the references of the included studies were checked to obtain a comprehensive list of studies. Study protocols were collected to consider future updates of the SR. We also obtained articles from other sources (e.g. hand search).

### Study selection

Two reviewers (AR, MM) independently screened titles and abstracts. When the abstracts seemed to meet the inclusion criteria, full texts were retrieved and assessed for inclusion. When needed, a third reviewer (ARG) was consulted to solve any disagreements. At the end of each search stage, the quality and completeness of the obtained studies were assessed and it was decided whether or not to proceed to the next stage of the search.

### Inclusion and exclusion criteria

Articles were assessed for inclusion regarding the type of study, age of participants, type of intervention, and clinical relevance of the outcomes.

The following articles were excluded: editorials, opinion papers, case reports, case series, narrative reviews, letters, qualitative studies, and OS which do not provide information regarding our outcomes. Articles not focussing on patient relevant outcomes were also excluded.

Table 1 displays details of the inclusion and exclusion criteria.

**Table 1:** Detailed inclusion and exclusion criteria regarding the type of study, age, intervention and outcomes

Criteria	Inclusion			Exclusion
Type of study	SR &MA	RCT	OS	editorials, opinion papers, case reports, case series, narrative reviews, letters, qualitative studies and observational studies which do not provide information of interest regarding adverse events
Age of participants	mean or median age ≥ 65 years, or subgroup analysis ≥ 65 years	≥ 80% of participants ≥ 65 years or a subgroup analysis ≥ 65 years		
Type of intervention	efficacy and/or safety of PAI as monotherapy or in combination with any other drug for the treatment or prevention of cerebral infarction, transient ischaemic attacks, peripheral artery occlusive disease and coronary disease or one of these indications versus placebo, no treatment, other drugs or a non-pharmacological intervention			focus on acute conditions (like exclusively acute treatment of myocardial infarction during the first hours)
Clinically relevant outcomes	quality of life, mortality, life expectancy, hospitalization, cognitive impairment or cognitive status, functional impairment or functional status, cardiovascular event including stroke, renal failure, composite end points including any of the above (extraction of individual outcomes will be done if reported by original studies), adverse drug event, bleeding			surrogate endpoints (like laboratory measurements or blood pressure)

*MA* meta-analysis, *OS* observational study, *RCT* randomised controlled trial, *SR* systematic review

**Table 2:** Characteristics of included SR/MA

Reference	Type of study	Aim	Population (number of studies, participants, mean age)	Treatment	Outcomes
Aguilar 2005 [49]	SR	To assess the efficacy and safety of long-term APT for primary prevention in stroke of patients with chronic non-valvular AF.	Studies *n* = 5, 1965 participants, mean age 70 years	Warfarin INR 2.8–4.2 vs ASA 75 mg/d vs placebo, ASA 125 mg/d vs placebo, warfarin INR 2–4.5 vs control and ASA 325 mg/d vs placebo	All strokes, ischaemic strokes, all disabling or fatal stroke, myocardial infarction, systemic emboli, all intracranial haemorrhage, major extracranial haemorrhage, vascular death and the composite of all stroke, myocardial infarction, vascular death and all-cause mortality
Aguilar 2007 [30]	SR	To characterize the relative effect of long-term oral anticoagulant treatment compared with antiplatelet therapy on major vascular events in patients with non-valvular AF and no history of stroke or TIA.	Studies *n* = 7, 9598 participants, mean age 64–75 years	Clopidogrel 75 mg/d + ASA 75-100 mg/d vs warfarin INR 2–3, ASA 75 mg/d vs warfarin INR 2.8–4.2 vs placebo, ASA 300 mg/d vs fixed-dose warfarin vs fixed-dose warfarin + ASA 300 mg/d vs adjusted-dose warfarin INR 2–3, ASA 100 mg/d vs fixed-dose VKA vs adjusted-dose VKA INR 2.6–3.5, triflusal 600 mg/d vs adjusted-dose VKA INR 2–3, ASA 150 mg/d vs warfarin INR 2.5–3.5 vs low-dose warfarin INR 1.1–1.6, ASA 325 mg/d vs warfarin INR 2–4.5, ASA 325 mg/d vs adjusted-dose warfarin INR 2–4.5	Primary outcome: all strokes; Secondary outcomes: ischaemic strokes, all disabling or fatal strokes, myocardial infarction, systemic emboli, all intracranial haemorrhages, major extracranial haemorrhages, vascular death, all-cause mortality
Andersen 2008 [43]	MA	To evaluate the efficacy of warfarin in preventing systemic embolism (embolism to limbs or viscera) in patients with AF	Studies *n* = 15, 16,058 participants, mean age 63.3–81.5 years	Warfarin vs placebo vs ASA 75 mg/d, warfarin vs placebo, warfarin vs ASA 325 mg/d, warfarin vs low-dose warfarin (INR 1.2–1.5) + ASA 325 mg/d, warfarin vs indobufen 200 mg/d, warfarin vs low-dose warfarin 1.25 mg/d, warfarin vs low-dose warfarin 1.25 mg/d vs low-dose warfarin + ASA 300 mg/d vs ASA 300 mg/d, warfarin vs ASA 150 mg/d vs low-dose warfarin (INR 1.1–1.6), warfarin vs low-dose warfarin (INR 1.5–2.1), warfarin vs ASA 150-160 mg/d, warfarin vs clopidogrel 75 mg/d + ASA 75-100 mg/d, warfarin vs ASA 300 mg/d, warfarin vs ASA 75 mg/d	Systemic embolism and major bleeding
Assiri 2013 [44]	MA	A mixed treatment comparison meta-analysis to evaluate direct and indirect treatment data including ASA, warfarin apixaban, dabigatran, edoxaban and rivaroxaban for the prevention of primary or secondary stroke in patients with AF	Studies *n* = 21, 80,906 participants, mean age 71 years	ASA vs warfarin vs placebo, ASA vs warfarin, warfarin vs placebo, ASA vs placebo, ASA + warfarin vs placebo, ASA + clopidogrel vs warfarin, dabigatran vs warfarin, ASA vs ASA + clopidogrel, edoxaban vs warfarin, apixaban vs warfarin, rivaroxaban vs warfarin	Any stroke or embolism, all-stroke, ischemic stroke, systemic embolism, vascular death, all-cause mortality, major and non-major bleeding, and intra-cranial haemorrhage
Baigent 2009 [28]	MA	To assess the benefits and risks in primary prevention; Identify risk factors for various outcomes in people in the primary prevention trials	Studies *n* = 6 primary prevention, 16 secondary prevention, 112,000 participants, subgroup ≥ 65 years	ASA 500 mg/d, ASA 325 mg/d vs placebo, ASA 75 mg/d vs warfarin vs placebo, ASA 75 mg/d vs placebo, ASA 100 mg/d vs control, ASA 100 mg/d vs placebo	Vascular events (myocardial infarction, stroke, death from vascular cause), major coronary event, any stroke, death from any cause, major extracranial bleeding
Cameron 2014 [46]	NMA	To examine the comparative efficacy and safety of antithrombotic treatments (apixaban, dabigatran, edoxaban, rivaroxaban and VKA at a standard adjusted dose (target international normalised ratio 2.0–3.0), ASA, ASA and clopidogrel) for non-valvular atrial fibrillation and among subpopulations	Studies *n* = 16, 82,314 participants, mean age 62–83, subgroup-analysis age ≥ 75 years	Dabigatran 150 mg twice daily vs dabigatran 110 mg twice daily vs adjusted-dose VKA, edoxaban 60 mg/d vs edoxaban 30 mg/d vs adjusted-dose VKA, ASA 100 mg/d + clopidogrel 75 mg/d vs adjusted-dose VKA, ASA 100 mg/d vs placebo, ASA 100 mg/d vs adjusted-dose VKA, adjusted-dose VKA vs placebo, ASA 100-300 mg/d vs placebo, ASA 100-300 mg/d vs adjusted-dose VKA, rivaroxaban 20 mg/d vs adjusted-dose VKA, apixaban 5 mg twice daily vs adjusted-dose VKA, dabigatran 150 mg twice daily 100-300 mg ASA vs adjusted-dose VKA, apixaban 2.5 mg twice daily vs apixaban 5 mg twice daily vs adjusted-dose VKA	Primary outcomes: all-cause stroke, systemic embolism, major bleeding
Cooper 2006 [42]	NMA	To identify different stroke prevention treatments for atrial fibrillation assessed in randomized controlled trials and to compare them within a single evidence synthesis framework	Studies *n* = 19, 17,833 participants, mean age 64–80.5 years	Warfarin INR 2–3 vs ximelegatran 72 mg/d, warfarin 1.25 mg/d + ASA 75 mg/d vs control, warfarin INR 2.2–3.5 vs warfarin INR 1.5–2.1, warfarin INR 1.2–1.5 vs control, warfarin prothrombin-time 1.2–1.5 vs placebo, warfarin INR 2–3 vs placebo, warfarin vs ASA 300 mg/d vs placebo, ASA 325 mg/d vs warfarin vs placebo, warfarin 1.25 mg/d vs warfarin 1.25 mg/d + ASA 300 mg/d vs ASA 300 mg/d vs warfarin INR 2.3, coumarin INR 2.5–3.5 vs coumarin INR 1.1–1.6 vs ASA 150 mg/d, indobufen 100 or 200 mg/d vs warfarin INR 2–3.5, warfarin INR 1.25 mg/d vs warfarin INR 2–3, warfarin INR 1.2–1.5 + ASA 325 mg/d vs warfarin INR 2–3, ASA 125 mg/d vs ASA 125 mg/d on alternate days vs control	Primary outcome: ischaemic stroke, major or fatal bleeding
Coleman 2012 [56]	MA	To identify the propensity difference between various AP and anticoagulation for stroke prevention in patients with AF to cause MGIB	Studies *n* = 16, 42,983 participants, mean age 65–75 years	Clopidogrel 75 mg/d + ASA 75-100 mg/d, ASA 75-100 mg/d vs dabigatran 110 mg BID vs dabigatran 150 mg BID vs adjusted-dose warfarin, ASA 150-200 mg/d vs control, adjusted-dose warfarin vs ximelagatran 36 mg BID, triflusal 600 mg/d vs adjusted-dose VKA vs adjusted-dose VKA + ASA 100 mg/d, adjusted-dose VKA + placebo vs adjusted-dose VKA + ASA 100 mg/d, adjusted-dose warfarin vs low-dose warfarin, adjusted-dose VKA vs low-dose VKA vs ASA 150 mg/d, adjusted-dose warfarin vs low-dose warfarin vs ASA 300 mg/d vs low-dose warfarin + ASA 300 mg/d, indobufen 200 mg BID vs adjusted-dose warfarin, low-dose warfarin + ASA 325 mg/d, adjusted-dose warfarin (INR 2–3 or 2–4.5) vs ASA 325 mg/d, adjusted-dose VKA vs ASA 300 mg/d vs placebo, adjusted-dose warfarin vs placebo, warfarin vs no treatment, adjusted-dose warfarin vs ASA 75 mg/d vs placebo	Major gastrointestinal bleeding
Connolly 2013 [61]	MA	To characterize the risk of subdural hematoma associated with antiplatelet therapy	Studies *n* = 9, 97,254 participants, mean age 57 years, subgroup ≥ 70 years	ASA 325 mg/d vs placebo, ASA 75 mg/d vs placebo, ASA 150-200 mg/d vs control, ASA 81-100 mg/d vs control, ASA 325 mg every other day vs placebo, ASA 50 mg/d + dipyridamol 400 mg/d vs placebo, ASA 75 mg/d vs placebo, ASA 100 mg every other day vs placebo, ASA 100 mg/d vs placebo	Subdural hematomas, intracerebral haemorrhage
Dogliotti 2014 [41]	NMA	To synthesise the evidence from trials using a multiple treatment comparison methods thereby permitting a broader comparison across multiple therapies	Studies *n* = 20, 79,808 participants, mean age 64–83	Adjusted-dose warfarin vs ASA vs placebo, adjusted-dose warfarin vs placebo, adjusted-dose VKA vs ASA vs placebo, adjusted-dose warfarin vs ASA, adjusted-dose coumarin vs ASA, ASA vs no treatment, adjusted-dose warfarin vs ASA + clopidogrel, ASA vs no treatment, clopidogrel + ASA vs ASA, adjusted-dose warfarin vs ASA, dabigatran 110 mg twice daily vs dabigatran 150 mg twice daily vs adjusted-dose warfarin, apixaban vs adjusted-dose warfarin, apixaban vs ASA, adjusted-dose warfarin vs rivaroxaban	Primary outcomes: stroke, composite of ischaemic stroke or systemic embolism, death from any cause, major bleeding
Gandhi 2015 [54]	MA	To compare Dual-antiplatelet Therapy to Mono-antiplatelet Therapy after Transcatheter Aortic Valve Implantation	Studies *n* = 4, 640 participants, mean age 82.2 years	ASA 80 mg/d + clopidogrel 75 mg/d or ticlopidin 500 mg BID vs ASA 75-160 mg/d, ASA 75 mg/d + clopidogrel 75 mg/d (300 mg loading-dose) vs ASA 75 mg/d or clopidogrel 75 mg/d, ASA 75 mg/d + clopidogrel 75 mg/d (300 mg loading-dose) vs ASA 75 mg/d (300 mg loading-dose), ASA 100 mg/d + clopidogrel 75 mg/d (300 mg loading-dose) vs ASA 100 mg/d	Primary outcome: combined end point of 30-day stroke, spontaneous myocardial infarction, all-cause-mortality, combined lethal and major bleeding. Secondary outcomes: 30-day major stroke, 30-day spontaneous myocardial infarction, 30-day all-cause mortality, 30-day combined lethal and major bleeding, 6-months major stroke, 6-months myocardial infarction, 6-months all-cause mortality, 6-months combined lethal and major bleeding
Halkes 2008 [56]	MA	To study the effect of combination therapy with ASA and dipyridamole (A + D) over ASAalone in secondary prevention after transient ischemic attack or minor stroke of presumed arterial origin and to perform subgroup analyses to identify patients that might benefit most from secondary prevention with A + D	Studies *n* = 5, 7612 participants, mean age 65 years	ASA + dipyridamol vs ASA, ASA + dipyridamol vs ASA vs dipyridamol vs placebo. ASA ranged from 50 mg/d-990 mg/d. Dipyridamol ranged from 150 mg/d-400 mg/d.	Death from all vascular causes, nonfatal stroke, nonfatal myocardial infarction
Hart 2007 [40]	MA	To characterize the efficacy and safety of antithrombotic agents for stroke prevention in patients who have atrial fibrillation, adding 13 recent randomized trials to a previous meta-analysis	Studies *n* = 29, 28,044 participants, mean age 71 years	Warfarin vs ASA vs placebo, warfarin vs control, warfarin vs placebo, LMWH vs control, warfarin vs ASA, warfarin vs low-dose warfarin + ASA, warfarin vs indobufen, ASA vs dipyridamol vs ASA + dipyridamol vs placebo, warfarin vs low-dose warfarin vs ASA vs low-dose warfarin + ASA, warfarin vs low-dose warfarin, warfarin vs low-dose warfarin vs ASA, ASA daily vs ASA every other day vs control, ASA vs placebo, warfarin vs warfarin, fluindione vs fluindione + ASA, ximelagatran vs warfarin, low-dose warfarin + ASA vs control, triflusal vs VKA vs triflusal + VKA, ximelagatran vs warfarin, ASA vs control, warfarin vs clopidogrel + ASA, dabigatran vs dabigatran +ASA vs warfarin	All strokes, ischaemic stroke, intracranial haemorrhage, all-cause mortality, major extracranial haemorrhage
Hart 1999 [39]	MA	To analyse the increased risk of intracranial haemorrhage when ASA is combined with Warfarin	Studies *n* = 16, 9874 participants, mean age 69–71 years	Warfarin INR 2.8–4.2 vs ASA 75 mg/d, ASA 300 mg/d vs warfarin INR 2–3, warfarin vs placebo + ASA 325 mg/d vs Placebo, ASA 325 mg/d vs warfarin 2–4.5, warfarin INR 1.2–1.5 + ASA 325 mg/d vs warfarin INR 2–3, warfarin INR 1.2–1.5 vs Control, warfarin vs placebo, warfarin 1.2–1.5 vs placebo, OAC INR 3–4.5 vs ASA 300 mg/d vs placebo, indobufen 100-200 mg vs warfarin INR 2–3.5, warfarin fixed-dose 1.25 mg/d vs warfarin INR 2–3, ASA 150 mg/d vs warfarin INR 2.5–3.5, LMWH vs control, ASA 125 mg/d vs ASA 125 mg/d on alternate days vs control, ASA 600 mg/d vs ASA 300 mg/d vs placebo	All strokes, ischaemic stroke, intracranial haemorrhage, all-cause mortality, major extracranial bleeding
He 1998 (JAMA) [29]	MA	To estimate the risk of haemorrhagic stroke associated with ASA treatment	Studies *n* = 16, 55,462 participants, mean age 63.7 years, subgroup ≥64 years	ASA 1200 mg/d vs placebo, ASA 1300 mg/d vs placebo, ASA 900 mg/d vs placebo, ASA 1000 mg vs ASA 1000 mg + 325 mg dipyridamol, ASA 1000 mg/d vs placebo, ASA 1500 mg/d vs placebo, ASA 160 mg/d vs streptokinase vs both vs placebo, ASA 500 mg/d vs placebo, ASA 325 mg/d vs placebo, ASA 75 mg/d vs warfarin vs placebo, ASA 325 mg/d vs warfarin vs placebo, ASA 75 mg/d vs placebo, ASA 300 mg/d vs placebo	Primary outcome: stroke. Secondary outcomes: myocardial infarction, cardiovascular disease mortality, all-cause mortality
Leonardi-Bee 2005 [55]	MA	To assess whether dipyridamole, given with or without ASA, reduced stroke in patients with previous ischemic cerebrovascular disease	Studies *n* = 7, 11,459 participants, mean age 65.4, subgroup age ≥ 65 years	Dipyridamol 100-200 mg/d vs control, dipyirdamol 20 mg/d + ASA 300 mg/d vs ASA 300 mg/d vs control, dipyridamol 75 mg/d + ASA 330 mg/d vs ASA 330 mg/d vs control, dipyridamol 75 mg/d + ASA 325 mg/d vs ASA 325 mg/d, dipyridamol 75 mg/d vs dipyridamol 75 mg/d + ASA 300 mg/d vs ASA 300 mg/d, dipyridamol 100 mg/d + ASA 50 mg/d vs dipyridamol 100 mg/d, dipyridamol 75 mg/d + ASA 330 mg/d vs control, dipyridamol 200 mg/d + ASA 25 mg/d vs ASA 25 mg/d vs dipyridamol 200 mg/d vs control	Primary outcome: composite of death from all vascular causes, fatal stroke, non-fatal myocardial infarction. Secondary outcomes: composite of death from all vascular causes or non-fatal stroke, all death, death from vascular causes, fatal and non-fatal stroke, fatal and non-fatal myocardial infarction
Lin 2015 [63]	NMA	To summarize and compare clinical and safety outcomes of oral antithrombotics for stroke prevention in AF in younger (65–74 years) and older (≥ 75 years) elderly	Studies *n* = 49, 897,748 participants, mean age 71 years, subgroup age ≥ 75 years	Dabigatran 150 mg vs dabigatran 110 mg vs warfarin, dabigatran 150 mg vs warfarin, rivaroxaban vs warfarin, apixaban vs warfarin, edoxaban vs warfarin, ASA vs warfarin, warfarin vs ASA + clopidogrel, warfarin vs ASA vs control, warfarin vs control, ASA vs control, apixaban vs ASA, ASA + clopidogrel vs ASA, warfarin vs ASA, warfarin vs ASA vs control, dabigatran 150 mg vs warfarin vs rivaroxaban, dabigatran vs rivaroxaban, dabigatran 150 mg vs dabigatran 110 mg vs rivaroxaban vs warfarin, dabigatran vs rivaroxaban vs warfarin, dabigatran vs warfarin, rivaroxaban vs warfarin, warfarin vs ASA vs ASA + clopidogrel	Primary outcomes: stroke, systemic embolism, major bleeding. Secondary outcomes: ischaemic stroke, all-cause mortality, intracranial bleeding, gastrointestinal bleeding
Lip 2006 [45]	MA	To compare the effectiveness of ASA, warfarin, and ximelagatran as thromboprophylaxis in patients with non-valvular atrial fibrillation	Studies *n* = 13, 14,423 participants, mean age 64–80 years	Warfarin INR 2.8–4.2 vs ASA 75 mg/d vs placebo, warfarin INR 1.5–2.7 vs placebo, warfarin INR 2–3 vs placebo, warfarin prothrombin time 1.3–1.8 vs placebo, warfarin INR 1.4–2.8 vs placebo, warfarin 2.5–4.0 vs placebo, warfarin INR 2.7 vs ASA 325 mg/d, warfarin INR 2.6 vs ASA 325 mg/d, warfarin INR 2–3 vs fixed low-dose warfarin + ASA 325 mg/d, warfarin INR 2–3 vs ASA 300 mg/d vs fixed low-dose warfarin vs fixed low-dose warfarin + ASA 300 mg/d, warfarin INR 2.5–3.5 vs ASA 150 mg/d vs fixed low-dose warfarin, warfarin INR 2–3 vs ASA 75-300 mg/d, warfarin INR 2–3 vs ximelagatran 72 mg/d, warfarin INR 2–3 vs ximelagatran 72 mg/d	Ischaemic stroke, systemic embolism, mortality, haemorrhage
Segal 2000 [38]	MA	To appropriate use of drugs to prevent thromboembolism in patients with AF involves comparing the patient’s risk of stroke and risk of haemorrhage. Summarize the evidence regarding the efficacy of the medicaments	Studies *n* = 11, 8690 participants, mean age 66–80 years	Warfarin vs placebo, ASA 325 mg/d vs warfarin, warfarin vs ASA 325 mg/d + low-dose warfarin, ASA 75 mg/d vs warfarin vs placebo, warfarin vs ASA 300 mg/d vs ASA 300 mg/d + low-dose warfarin, warfarin vs ASA 300 mg/d vs placebo, warfarin vs indobufen 200 mg, anti-factor Xa vs placebo	Stroke, major haemorrhage, minor haemorrhage, total mortality
Taylor 2001 [37]	MA	To examine the benefits and risks of long term anticoagulation (warfarin) compared with antiplatelet treatment (ASA/indoprofen) in patients with non­rheumatic atrial fibrillation	Studies *n* = 6, 3298 participants, mean age 64–80 years	ASA 75 mg/d vs warfarin INR 2.8–4.2, ASA 325 mg/d vs warfarin INR 2–4.5, ASA 300 mg/d vs warfarin INR 2–3, indoprofen 400 mg/d vs warfarin INR 2–3.5, ASA 150 mg/d vs warfarin INR 2.5–3.5	Fatal and non-fatal cardiovascular events, fatal and major non-fatal bleeding events
Warkentin 2012 [57]	MA	To provide a pooled estimate of the bleeding risk from randomized controlled trials RCTs comparing warfarin and ASA at the dose ranges recommended in evidencebased guidelines	Studies *n* = 8, 2948 participants, mean age 62–83 years, subgroup age ≥ 70 years	ASA 80 mg/d vs warfarin INR 2–2.5, ASA 100 mg/d vs warfarin INR 2–3, ASA 300 mg/d vs warfarin INR 2–3, ASA 75 mg/d vs warfarin INR 2–3, ASA 325 mg/d vs warfarin INR 2–3, ASA 162 mg/d vs warfarin INR 2–3.5	Major bleeding
Zhou 2012 [52]	MA	To evaluate the benefits and harms of combined ASA and clopidogrel therapy on major cardiovascular outcomes	Studies *n* = 7, 48,248 participants, mean age 61.7–71 years	Clopidogrel 75 mg/d + ASA 75-325 mg/d vs ASA 75-325 mg/d, clopidogrel 75 mg/d + ASA 100-200 mg/d vs ASA 100-200 mg/d, clopidogrel 75 mg/d + ASA 75-100 mg/d vs ASA 75-100 mg/d, clopidogrel 75 mg/d + ASA 162 mg/d vs ASA 162 mg/d, clopidogrel 75 mg/d + ASA 75-162 mg/d vs ASA 75-162 mg/d, clopidogrel 75 mg/d + ASA 81-325 mg/d vs ASA 81-325 mg/d, clopidogrel 75 mg/d + ASA 75 mg/d vs clopidogrel 75 mg/d	Major cardiovascular events, myocardial infarction, stroke, mortality, major bleeding events, other adverse reaction

*AF* atrial fibrillation, *APT* Anti-platelet therapy, *ASA* acetylsalicylic acid, *BID* twice a day, *INR* international normalized ratio, *LMWH* low molecular weight heparin, *MA* meta-analysis, *MGIP* major gastrointestinal bleeding, *NMA* network-meta-analysis, *OAC* oral anticoagulation, *RCT* randomized controlled trials, *SR* systematic review, *TIA* transient ischemic attack, *VKA* Vitamin K Antagonist

**Table 3:** Characteristics of included RCT

Reference	Type of study	Aim	Treatment	Sample size and amount of older participants	Follow-up	Outcomes
Britton 1987 [58]	RCT	To study the effectiveness of high-dose ASA after cerebral infarction	ASA 1.5 g/d vs placebo	*N* = 505, mean age 68 years	2 years	Primary outcomes: Recurrent stroke or death. Secondary outcomes: myocardial infarction, TIA
Diener 2004 [53]	RCT	To assess whether addition of ASA to clopidogrel could have a greater benefit than clopidogrel alone in prevention of vascular events with potentially higher bleeding risk	ASA 75 mg/d + clopidogrel 75 mg/d vs placebo + clopidogrel 75 mg/d	*N* = 7599, placebo + clopidogrel: mean age 66.1 years, 54% older than 65 years. ASA + clopidogrel: mean age 66.5 years, 56% older than 65 years. Subgroup analysis age ≥ 65 years	18 months	Primary outcomes: composite of ischaemic stroke, myocardial infarction, vascular death or rehospitalisation for an acute ischaemic event. Secondary outcomes: Individual and various combinations of each of the outcomes forming the primary endpoint, any death, any stroke
EAFT 1993 [34]	RCT	To assess the preventive benefit of anticoagulation or ASA in patients with recent transient ischaemic attack or minor ischaemic stroke	ASA 300 mg/d vs OAC INR 2.5–4.0 vs placebo	*N* = 1007, OAC: mean age 71 years. 80% older than 65 years. ASA: mean age 73 years, 84% older than 65 years. Placebo: mean age 73 years, 84% older than 65 years	2.3 years	Primary outcomes: death from vascular disease, non-fatal stroke (including haemorrhage), non-fatal myocardial infarction or systemic embolism. Secondary outcomes: death from all causes, all strokes, major thromboembolic events
Huynh 2001 [36]	RCT	To test the hypothesis that moderate-intensity warfarin either alone or in combination with low-dose ASA will be more effective than ASA alone for the secondary prevention of coronary events in patients with previous CABG	ASA 80 mg/d + placebo, warfarin INR 2–2.5 + placebo, ASA 80 mg/d + warfarin INR 2–2.5	*N* = 135, ASA + placebo: mean age 68, 61% older than 65 warfarin + placebo: mean age 67, 57% older than 65, ASA + warfarin: mean age 66, 53% older than 65 years. Subgroup analysis age ≥ 65 years	12 months	Primary outcomes: composite end point of any-cause death, myocardial infarction, unstable angina requiring a new hospitalization. Secondary outcome: performance of reperfusion procedure (either percutaneous or open chest)
Ikeda 2014 [35]	RCT	To determine whether daily low-dose ASA reduces the incidence of cardiovascular events in older Japanese patients with multiple atherosclerotic risk factors	ASA 100 mg/d vs control	*N* = 14,464, ASA: mean age 70.6, 82% older than 65. Control: mean age 70.5, 81% older than 65	5.02 years	Primary outcome: composite of death from cardiovascular causes, non-fatal stroke and non-fatal myocardial infarction. Secondary outcomes: composite of primary outcomes + TIA, angina pectoris and arteriosclerotic disease requiring surgery or intervention; death from cardiovascular causes, non-fatal stroke, non-fatal myocardial infarction, TIA, angina pectoris, arteriosclerotic disease requiring surgery or intervention, serious extracranial haemorrhage
Kjeldsen 2000 (HOT) [31]	RCT	To study the relationship between three levels of target diastolic blood pressure and cardiovascular events in hypertensive patients and to examine the effects of 75 mg ASA daily versus placebo	ASA 75 mg/d	*N* = 18,790; men: mean age 60.8 years, 28% older than 65 years. Women: mean age 62.3 years, 36% older than 65 years. Subgroup analysis age ≥ 65 years	3.8 years	Major CV events, MI, Stroke CV mortality, total mortality
Liu 2014 [47]	RCT	To compare the therapeutic warfarin and ASA efficacies for treatments of atrial fibrillation complicated with stable coronary heart disease	Warfarin INR 1.6–2.5, ASA 100 mg/d	*N* = 101, warfarin: mean age 84.8 years, ASA: mean age 84.4 years. 100% older than 65 years	2 years	Primary outcome: ischaemic stroke, systemic embolism. Secondary outcomes: non-fatal myocardial infarction and all causes of death
Ogawa 2008 [33]	RCT	To examine the efficacy of low-dose ASA for the primary prevention of atherosclerotic events in patients with type 2 diabetes	ASA 81–100 mg/d vs control	*N* = 2539, mean age 65 years. ASA: 50% older than 65 years. Non-ASA group: 46% older than 65 years. Subgroup analysis age ≥ 65 years	4.37 years	Primary outcomes: Any atherosclerotic event (composite endpoint of sudden death, death from coronary, cerebrovascular, and aortic causes, non-fatal acute myocardial infarction, unstable angina, newly developed exertional angina, non-fatal ischaemic and haemorrhagic stroke, transient ischaemic attack, non-fatal aortic and peripheral vascular disease). Secondary outcomes: each primary endpoint and combinations of primary endpoints, death from any cause
Silagy 1993 [32]	RCT	To investigate the incidence of adverse effects resulting from the use of regular low-dose ASA in an otherwise healthy elderly population	ASA 100 mg/d vs placebo	*N* = 400, mean age 73 years, 100% older than 65 years	12 months	Adverse events (gastrointestinal symptoms, gastrointestinal bleeding, easy bruising, nose bleeds), haematologic parameters
Uchiyama 2016 [50]	RCT	To evaluate the effect of ASA on the risk of stroke and intracranial haemorrhage in the Japanese Primary Prevention Project.	ASA 100 mg/d vs control	*N* = 14,464, ASA: mean age 70.6 years, 82% older than 65 years. No ASA: 70.5 years, 81% older than 65 years	5.02 years	Primary outcomes: composite of death from cardiovascular causes (including fatal myocardial infarction, fatal stroke, and other cardiovascular death), non-fatal stroke and non-fatal MI. Secondary outcomes: composite of the same events as the primary end points plus TIA, angina pectoris and atherosclerotic disease requiring surgery or intervention. Death from cardiovascular disease, death from nonvascular causes, non-fatal stroke, non-fatal MI, TIA, angina pectoris, atherosclerotic disease requiring surgery or intervention, serious extracranial haemorrhage requiring transfusion or hospitalization.
Wiviott 2007 [51]	RCT	To compare prasugrel with clopidogrel for the prevention of thrombotic complications in patients with an acute coronary syndrom and scheduled percutaneous coronary intervention	Prasugrel 60 mg loading-dose, 10 mg daily maintenance dose vs clopidogrel 300 mg loading-dose, 75 mg daily maintenance	*N* = 13,608, mean age 61 years. Subgroup analysis age ≥ 65 years.	6–15 months	Primary outcomes: composite of death from cardiovascular causes, non-fatal myocardial infarction, or non-fatal stroke. Secondary outcomes: stent thrombosis and a composite of death from cardiovascular causes, non-fatal myocardial infarction, non-fatal stroke, or rehospitalisation due to a cardiac ischaemic event. Safety outcomes: major bleeding not related to coronary-artery bypass grafting, life threatening bleeding not related to coronary-artery bypass grafting, major and minor bleeding

*ASA* acetylsalicylic acid, *BID* twice a day, *CABG* coronary artery bypass graft, *CV* cardiovascular, *INR* international normalized ratio, *MI* myocardial infarction, *OAC* oral anticoagulation, *RCT* randomised controlled trial, *TIA* transient ischemic attack

**Table 4:** Characteristics of included observational studies

Reference	Type of study	Aim	Treatment	Sample size and number of older participants	Follow-up	Outcomes
Burton 2006 [48]	Regional cohort-study	To measure the complication rates and adequacy of warfarin control in a cohort of patients with atrial fibrillation managed in primary care and to compare them with published data from controlled trials and community patients with atrial fibrillation not receiving warfarin.	ASA, warfarin INR 2–3, no antithrombotic therapy	*N* = 601, mean age 77 years. Subgroup analysis age ≥ 75 years.	5 years	Antithrombotic treatment, stroke or TIA, bleeding complications, death
Sam 2004 [59]	Community-based observational cohort-study	To determine the prevalence of warfarin and ASA use in atrial fibrillation.	ASA, warfarin, control	*N* = 393, men: mean age 72.5 years, women: mean age 79 years.	14 years	ASA and warfarin use, bleeding complication, cardiovascular events

*ASA* acetylsalicylic acid, *MI* myocardial infarction, *INR* international normalized ratio, *TIA* transient ischaemic attack

**Table 5:** Quality appraisal SR/MA

Author, year	‘A priori’ Design	Duplicate study selection and data extraction	Comprehensive literature search performed	Status of publication used as an inclusion criterion	List of included and excluded studies provided	Characteristics of the included studies provided	Scientific quality of included studies assessed and documented	Scientific quality of included studies used appropriately in formulating conclusions	Appropriate methods to combine the findings of studies	Assessment of the likelihood of publication bias	Conflict of interest stated
Aguilar 2005 [49]	✓	✓	✓	**u**	✓	✓	✓	✓	✓	**✗**	✓
Aguilar 2007 [30]	✓	✓	✓	**u**	✓	✓	✓	✓	✓	**✗**	✓
Andersen 2008 [43]	✓	✓	✓	**✗**	✓	✓	✓	✓	**n/a**	✓	✓
Assiri 2013 [44]	✓	**u**	✓	**✗**	**✗**	✓	**u**	**u**	✓	**✗**	✓
Baignent 2009 [28]	✓	**✗**	✓	**✗**	✓	✓	**✗**	**✗**	✓	**✗**	✓
Cameron 2014 [46]	✓	✓	✓	✓	✓	✓	**✗**	**✗**	✓	**✗**	✓
Coleman 2012 [56]	✓	✓	✓	✗	✓	✓	✓	✓	✓	✓	**✗**
Connolly 2013 [61]	✓	✓	**✗**	✓	✓	✓	**✗**	**✗**	✓	**✗**	**✗**
Cooper 2006 [42]	✓	**✗**	✓	**✗**	**✗**	✓	**✗**	**u**	**✗**	**✗**	**✗**
Dogliotti 2014 [41]	✓	✓	✓	**✗**	**✗**	✓	**✗**	✓	**✗**	**✗**	✓
Gandhi 2015 [54]	✓	✓	✓	✓	✓	✓	✓	**u**	✓	✓	✓
Halkes, 2008 [56]	✓	**n/a**	**n/a**	**✗**	✓	✓	**✗**	**✗**	✓	**✗**	✓
Hart 1999 [39]	✓	✓	✓	**✗**	**✗**	✓	**✗**	**u**	✓	**✗**	**✗**
Hart 2007 [40]	✓	✓	✓	✓	✓	✓	✓	✓	✓	**✗**	✓
He 1998 [29]	✓	✓	**✗**	**✗**	✓	✓	**✗**	**✗**	✓	**✗**	✓
Leonardi-Bee 2005 [55]	✓	**n/a**	✓	✓	✓	✓	✓	✓	**✗**	**✗**	✓
Lin 2015 [57]	✓	✓	✓	✓	✓	✓	✓	**✗**	**✗**	✓	✓
Lip 2006 [45]	✓	**✗**	✓	**✗**	**✗**	✓	**✗**	**u**	✓	✓	✓
Segal 2000 [38]	✓	✓	✓	**✗**	✓	✓	✓	✓	✓	**✗**	✓
Taylor 2001 [37]	✓	**u**	✓	✓	**✗**	✓	**u**	✓	✓	✓	✓
Warkentin 2012 [57]	✓	✓	✓	**u**		✓	✓	✓	✓	**n/a**	✓
Zhou 2012 [52]	✓	✓	✓	✓	✓	✓	✓	✓	✓	✓	✓

**Table 6:** Quality appraisal RCT

Reference	Random sequence generation	Allocation concealment	Blinding of participants/personnel	Blinding of outcome assessment	Incomplete outcome data	Selective Reporting	Other bias
Britton 1987 [58]	LR	LR	LR	LR	LR	LR	UR
Diener 2004 (MATCH) [53]	LR	LR	UR	UR	LR	LR	LR
EAFT 1993 [34]	LR	LR	HR	LR	LR	LR	UR
Huynh 2001 [36]	UR	UR	LR	UR	LR	LR	HR
Ikeda 2014 [35]	LR	LR	HR	LR	LR	LR	LR
Kjeldsen 2000 [31]	LR	UR	UR	UR	LR	LR	HR
Liu 2014 [47]	UR	UR	HR	UR	LR	LR	LR
Ogawa 2008 [33]	LR	LR	HR	LR	LR	HR	UR
Silagy 1993 [32]	UR	UR	UR	UR	LR	LR	LR
Uchiyama 2016 [50]	LR	LR	HR	LR	UR	LR	LR
Wiviott 2007 [51]	UR	UR	UR	UR	LR	LR	LR

Note: *HR* high risk, *LR* low risk, *UR* unclear risk

**Table 7:** Quality appraisal OS

Reference	Study addressed a clearly focused issue	Authors used an appropriate method to answer their question	Cases/cohort were/was recruited in an acceptable way	Controls were selected in an acceptable way	The exposure was accurately measured to minimize bias	The outcome was accurately measured to minimize bias	The authors identified all important confounding factors	The follow up of subjects was complete enough	The follow up of subjects was long enough	Results can be applied to the local population
Sam 2004 [59]	✓	✓	✓	✓	✓	**✗**	**✗**	✓	✓	**✗**
Burton 2006 [48]	✓	✓	✓	✓	✓	**✗**	**✗**	✓	✓	✓

Note: ✓: yes, **✗**: no

**Table 8:** Quality, strength and evidence-base of the developed recommendations

Indication	Recommendations	Strength of the recommendation	Quality of the evidence	Evidence base
Primary prevention CVD (ASA not recommended)	It is suggested to discontinue ASA for primary prevention of CVD in adults without diabetes because there is uncertainty about the risk/benefit ratio: The risk of haemorrhagic stroke, major gastrointestinal and extracranial bleeds may be increased, there is less confidence regarding its benefits in decreasing vascular events in adults aged 65 and older.	Weak	Low	[28–35]
ASA in the secondary prevention of CVD	No stop recommendation developed			[36]
ASA in the primary prevention of stroke a) with AF b) without AF	With AF: It is suggested to discontinue ASA for the primary prevention of stroke in older adults with atrial fibrillation (including adults older than 75 years) and consider the use of oral anticoagulants instead. Oral anticoagulants are more beneficial than ASA in preventing cardiovascular events and these benefits appear to apply to older people, while the risk of bleeding appears to be similar for both treatments. Without AF: No stop recommendation developed	Weak	Low	With AF: [30, 37–49, 56] Without AF: [50]
ASA in the secondary prevention of stroke a) with AF b) without AF	No recommendation developed			With AF: [34, 39, 40] Without AF: [52]
ADP-receptor inhibitors in secondary prevention of cardiovascular disease	It is recommended to discontinue ASA in adults at high-risk of vascular events with recent transient ischaemic attack or ischaemic stroke who are also taking clopidogrel and who do not have another indication for dual therapy (e.g. first year after acute coronary syndrome, first year after elective drug eluting coronary stenting, aortic valve replacement, carotid stenting or complications of severe lower limb ischaemia despite ASA therapy) because the combination of ASA and clopidogrel compared with clopidogrel alone increases the risk of bleeding complications and may not be beneficial in reducing vascular events, especially in the subgroup of adults aged 65 years or older.	Strong	Moderate	[51, 52]
ADP- receptor inhibitors in the secondary prevention of stroke and/or transient ischemic attack	No recommendation developed			[52–54]
Dipyridamol in the secondary prevention of stroke	No recommendation developed			[55, 56]

Note: *MA* meta-analysis, *RCT* randomised controlled trial, *SR* systematic review

**Table 9:** Characteristics of participants in included SR/MA

Reference	Setting/country/ethnicity	Male sex	Age	Comorbidity	Number of coincident medications	Functional status/Frailty level	Cognitive status
Aguilar 2005 [49]	Denmark, Spain, USA	62%	Mean age 70 years	Not reported	Not reported	Not reported	Not reported
Aguilar 2007 [30]	Asia, Austria, Denmark, Greece, Netherlands, South America, South Africa, Spain, USA	Ranged from 45 to 66%	Mean age 64–75 years	Not reported	Not reported	Not reported	Not reported
Andersen 2013 [43]	Not reported	Not reported	Mean age 63.3–81.5 years	Most of the participants had at least one risk factor for cerebral embolism: previous MI, hypertension, diabetes mellitus, heart failure and/or stroke	Not reported	Not reported	Not reported
Assiri 2013 [44]	Not reported	58.7%	Mean age 71 years	Not reported	Not reported	Not reported	Not reported
Baigent 2009 [28]	Asia, Italy, UK, USA	Not reported	Subgroup ≥65 years	Not reported	Not reported	Not reported	Not reported
Cameron 2014 [46]	America, Asia, Canada, Denmark, Japan, UK,	Not reported	Mean age 62–83 years	Prior stroke 3%–55%	Not reported	Not reported	Not reported
Coleman 2012 [56]	Denmark, France, Italy, Japan, Netherlands, Spain, USA	Ranged from 41%–100%	Mean age 65–75 years	Not reported	Not reported	Not reported	Not reported
Cooper 2006 [42]	Asia, Australia, Canada, Denmark, Italy, Japan, Netherlands, Spain, Sweden, USA	Ranged from 32%–100%	Mean age 64–80.5 years	Previous stroke 0–100%	Not reported	Not reported	Not reported
Connolly 2013 [61]	Japan, Sweden, USA	41%	Mean age 57 years, subgroup ≥70 years	Not reported	Not reported	Not reported	Not reported
Dogliotti 2014 [41]	Canada, Japan, UK, USA	Ranged from 46 to 100%	Mean age 64–83 years	Hypertension 31.5–90.5%, diabetes mellitus 4–40%, previous myocardial infarction 8.6–19%, heart failure 5–69.5%, previous TIA/stroke 3.7–70%	Not reported	Not reported	Not reported
Gandhi 2015 [54]	France, Italy, UK	43%	Mean age 82.2 years	Hypertension 99%, dyslipidemia 52%, coronary heart disease 47%, diabetes mellitus 24%, atrial fibrillation 18%, chronic kidney disease 8.1%, previous myocardial infarction 13%	Not reported	Not reported	Not reported
Halkes 2008 [56]	Canada, France, USA	Ranged from 63 to 64%	Mean age 65 years	Hypertension 58%, diabetes mellitus 16–17%, ischaemic heart disease 25%	Not reported	Not reported	Not reported
Hart 2007 [40]	China, Japan, USA	75%	Mean age 71 years	Not reported	Not reported	Not reported	Not reported
Hart 1999 [39]	Canada, UK, USA	Ranged from 62 to 71%	Mean age 69–71 years	Hypertension 45–46%, previous TIA/stroke 20–40%	Not reported	Not reported	Not reported
He 1998 (JAMA) [29]	Australia, Canada, Denmark, France, Sweden, UK. USA, 99% white	86%	Mean age 59, subgroup ≥64 years	Hypertension 24%, hyperlipidaemia 11%, smoking 21%	Not reported	Not reported	Not reported
Leonardi-Bee 2005 [55]	Canada, France, Italy, Spain, USA	60%	Mean age 65.4	Not reported	Not reported	Not reported	Not reported
Liang Lin 2015 [63]	Asia, Canada, China, Denmark, Hong Kong, Israel, Japan, Netherlands, Spain, Sweden, UK, USA	NRSs 56%, RCTs 62%	Mean Age 71.5 years, subgroup-analysis age ≥ 75 years	Hypertension 17–94%, chronic heart failure 1–70%, diabetes mellitus 3–45%, prior TIA/stroke 3–55%	Not reported	Not reported	Not reported
Lip 2006 [45]	Canada, Denmark, Italy, Netherlands, USA	Not reported	Mean age 64–80 years	Not reported	Not reported	Not reported	Not reported
Segal 2000 [38]	Canada, Denmark, Netherlands, Sweden, USA	Ranged from 24%–100%	Mean age 66–80 years	Diabetes mellitus 8%–32%, congestive heart failure 9%–71%, hypertension 32%–58%	Not reported	Not reported	Not reported
Taylor 2001 [37]	Denmark, Italy, Netherlands, USA	Not reported	Mean age 64–80 years	Not reported	Not reported	Not reported	Not reported
Warkentin 2012 [57]	Canada, Denmark, Greece, UK, Spain, USA	Ranged from 47 to 89%	Mean age 62–83 years	Not reported	Not reported	Not reported	Not reported
Zhou 2012 [52]	Canada, USA	Ranged from 58.2–89.4%	Mean age 61.7–71 years, subgroup-analysis age ≥ 65 years	Not reported	Not reported	Not reported	Not reported

Note: *NRS* Numerical Rating Scale, *RCT*: randomized controlled trial, *TIA*: transient ischaemic attack

**Table 10:** Characteristics of participants in included RCTs

Reference	Setting/country/ethnicity	Male sex	Age	Comorbidity	Number of coincident medications	Functional status/frailty level	Cognitive status
Britton 1987 [58]	Sweden	ASA: 67%, placebo: 58%	Mean age 68 years, subgroup analysis age ≥ 68 years	ASA/placebo: hypertension 48%/45%, diabetes mellitus 15%/18%, smoking 48%/57%, hyperlipidaemia 2%/3%, angina 20%/15%, myocardial infarction 11%/9%, heart failure 15%/17%, atrial fibrillation 6%/8%, claudication 9%/8%, previous TIA 8%/8%, previous cerebral infarction 6%/6%, previous other strokes 5%/5%	Not reported	Capable of walking by themselves and without aphasia 61%, severely disabled 39%	Not reported
Diener 2004 [53]	28 countries	Placebo: 63%, intervention: 63%	Placebo + clopidogrel: mean age 66.1 years, 54% older than 65 years. ASA + clopidogrel: mean age 66.5 years, 56% older than 65 years, subgroup analysis age ≥ 65 years	Placebo + clopidogrel/ASA + clopidogrel: hypertension 78%/78%, diabetes mellitus 68%/68%, hypercholesterinaemia 57%/56%, smoking 47%/48%, previous ischaemic stroke 26%/27%, previous TIA 19%/19%, previous myocardial infarction 5%/5%, angina pectoris 12%/13%, symptomatic peripheral arterial disease 10%/10%	Not reported	Not reported	Not reported
EAFT 1993 [34]	Israel	OAC: 55%, ASA: 59%, placebo: 53%	OAC: mean age 71 years, 80% older than 65 years. ASA: mean age 73 years, 84% older than 65 years, placebo: mean age 73 years, 84% older than 65 years	Warfarin/ASA/placebo: Multiple strokes in the year prior to randomisation 19%/22%/24%, hypertension 43%/49%/47%, diabetes mellitus 12%/13%/13%, hypercholesterinaemia 12%/10%/7%, smoking 19%/20%/18%, angina pectoris 11%/11%/11%, myocardial infarction 7%/7%/9%, congestive heart failure 8%/11%/12%, minor stroke 8%/8%/6%	Not reported	Not reported	Not reported
Huynh 2001 [36]	Canada	ASA + placebo: 82.2%, warfarin + placebo: 86.4%, ASA + warfarin: 70.7%	ASA + placebo: mean age 68 years, 61% older than 65 years. Warfarin + placebo: mean age 67 years, 57% older than 65 years. ASA + warfarin: mean age 66 years, 53% older than 65 years, subgroup analysis age ≥ 65 years	ASA + placebo/warfarin + placebo/warfarin + ASA: hypertension 34.8%/37.8%/38.6%, prior myocardial infarction 56.5%/62.2%/72.7%, current smoking 17.8%/31.1%/20.5%, hyperlipidaemia 43.5%/62.2%/68.2%, diabetes mellitus 17.4%/15.6%/25%	ASA + placebo/warfarin + placebo/warfarin + ASA: β-blockers 71.7%/57.8%/70.5%, calcium antagonists 52.2%/55.6%/54.5%, nitrates 63%/64.4%/75%, lipid-lowering agents 37%/42.2%/36.4%, ACE inhibitors 15.3%/35.6%/22.7%, antiarrhythmic agents 2.2%/6.8%/4.6%, diuretics 11%/26.7%/34.1%	Not reported	Not reported
Ikeda 2014 [35]	Japan	ASA 42.3%, control 42.4%	ASA: mean age 70.6 years, 82% older than 65 years. Control: mean age 70.5 years, 81% older than 65 years	ASA/control: BMI >25 36.6%/35.9%, hypertension 84.9%/84.8%, dyslipidaemia 72%/71.8%, diabetes mellitus 33.9%/33.9%, smoking 13.3%/12.9%	Not reported	Not reported	Not reported
Kjeldsen 2000 (HOT) [31]	26 countries	53%	Men: mean age 60.8 years, 28% older than 65 years. Women: mean age 62.3 years, 36% older than 65 years, subgroup analysis age ≥ 65 years	Men/women: smoking 21.2%/10%, diabetes mellitus 7.8%/8.2%, previous cardiovascular disease 9.8%/7.4%	Men/women: calcium antagonists 45.2%/37.9%, β-blockers 28.3%/28.2%, ACE-inhibitors 39.3%/37.2%, diuretics 29.8%/36.4%	Not reported	Not reported
Liu 2014 [47]	China	Warfarin: 60.8%, ASA: 60.0%	Warfarin: mean age 84.8 years. ASA: mean age 84.4 years, 100% older than 65 years	Warfarin/ASA: hypertension 39.2%/38%, diabetes mellitus 21.6%/22%, smoking 25.5%/24%, previous myocardial infarction 15.7%/14%, angina pectoris 84.3%/86%	Warfarin/ASA: β-blockers 7.8%/6%, statins 11.2%/16%, ACE-inhibitors or ARB 9.8%/12%	Not reported	Not reported
Ogawa 2008 [33]	Japan	ASA: 56%, non-ASA: 53%	ASA: mean age 65 years, 50% older than 65 years. NonASA group: mean age 64 years, 46% older than 65 years, subgroup analysis age ≥ 65 years	ASA/non-ASA: smoking 23%/19%, hypertension 59%/57%, dyslipidaemia 54%/52%, diabetes mellitus 100%/100%	ASA/non-ASA: sulfonylureas 58%/56%, α-glukosidase-inhibitors 33%/32%, biguanides 13%/15%, insulin 13%/13%, thiazolidines 5%/5%, calcium antagonists 35%/34%, angiotensin-II antagonists 21%/21%, ACE-inhibitors 14%/15%, β-blockers 6%/7%, α-blockers 4%/3%, statins 26%/26%	Not reported	Not reported
Silagy 1993 [32]	Australia, Germany, USA	ASA: 48.6%, placebo: 49.6%	Mean age 73 years, 100% older than 65 years	ASA/placebo: smoking 4.5%/7.1%	Not reported	Not reported	Not reported
Uchiyama 2016 [50]	Japan	ASA: 42.3% no ASA: 42.4%	ASA: mean age 70.6 years, 82% older than 65 years. No ASA: 70.5 years, 81% older than 65 years	ASA/no ASA: hypertension 84.9%/84.8%, dyslipidaemia 72%/71.8%, diabetes mellitus 33.9%/33.9%, BMI >25 36.6%/35.9%, smoking 13.3%/12.9%	Not reported	Not reported	Not reported
Wiviott 2007 [51]	30 countries	Prasugrel: 75%, clopidogrel 73%	Mean age 61 years, 13% older than 75 years, subgroup analysis age ≥ 65 years	Prasugrel/clopidogrel: hypertension 64%/64%, hypercholesterolemia 56%/56%, diabetes mellitus 23%/23%, smoking 38%/38%, previous myocardial infarction 18%/18%, previous CABG 8%/7%	Prasugrel/clopidogrel: heparin 66%/65%, LMWH 9%/8%, bivalirudin 3%/3%, glycoprotein IIb/IIIa–receptor antagonist 54%/55%, ACE inhibitors 76%/75%, β-blockers 88%/88%, statin 92%/92%, calcium antagonists 18%/17%, ASA 99%/99%	Not reported	Not reported

Note: *ASA* acetylsalicylic acid, *BMI* Body Mass Index, *CABG* coronary artery bypass graft, *LMWH* low molecular weight heparin, *OAC* oral anticoagulation, *TIA* transient ischaemic attack

**Table 11:** Characteristics of participants in included observational studies

Reference	Country	Male sex	Age	Comorbidity	Number of coincident medications	Functional status/frailty level	Cognitive status
Burton 2006 [48]	Scotland	51%	Mean age 77 years, subgroup analysis age ≥ 75 years	Not reported	Not reported	Not reported	Not reported
Sam 2004 [59]	USA	49.8%	Men: mean age 72.5 years. Women: mean age 79 years, 100% older than 65 years	ASA/warfarin/none: congestive heart failure 14%/22%/65%, previous myocardial infarction 20%/18%/62%, stroke 25%/28%/46%, diabetes mellitus 25%/23%/53%, hypertension 21%/21%/58%, alcohol use 19%/26%/55%	Not reported	Not reported	Not reported

Note: *ACE* angiotensin-converting-enzyme, *ASA* acetylsalicylic acid, *AT* Angiotensin, *NSAID* nonsteroidal anti-inflammatory drugs

### Data extraction and quality appraisal

Data extraction and quality appraisal were performed using piloted forms. One reviewer did data extraction and quality appraisal and a second reviewer checked the forms for completeness and accuracy. A third reviewer was used in cases of disagreement. Four reviewers (AR, CS, MM, MK) participated at this stage of the SR. Data extracted included the specific drugs and dosages, study methods, time to follow-up, characteristics of the participants, outcomes and results. The quality of the included studies was assessed using specifically validated assessment tools for each type of study design: for SR and MA the AMSTAR appraisal tool [20, 21] and for clinical trials the Cochrane Collaboration’s tool for assessing risk of bias [22]. For observational studies a selection of questions from the critical appraisal skills programme (CASP) was used [23, 24].

### Development of recommendations

A document containing a summary of all included studies, emphasising the risks and benefits of PAI was developed. This document and the quality of the study provided the basis for the development of recommendations on the discontinuation of PAI in older adults with cerebrovascular disease, peripheral artery occlusive disease, and coronary disease. Recommendations were judged regarding strength and quality of the evidence using the Grading of Recommendations Assessment Development and Evaluation (GRADE) methodology [25–27]. The final recommendations were worded following a standardised scheme clarifying strength and quality. Four reviewers (ARG, AS, IK, MM) were involved in the development and approval of the recommendations.

## Results

### Literature search and inclusion of studies

**Fig. 1:**
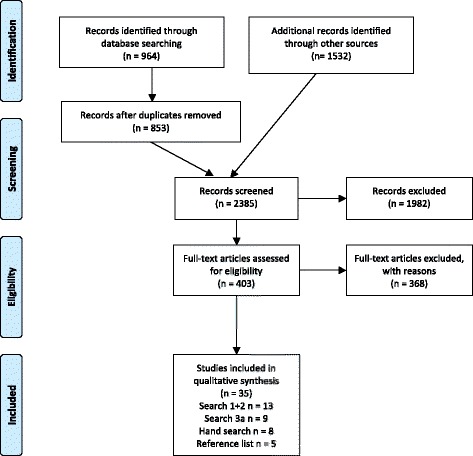
Preferred Reporting Items for Systematic Reviews and Meta-Analyses (PRISMA) flow diagram

Figure 1 displays the identification process of studies for inclusion in the SR in a PRISMA flow-chart. Searches 1, 2 and 3a were performed. The research team decided not to perform search 3b for the reasons described above.

There were 964 references identified in the electronic databases during search 1 and 2. After the exclusion of all duplicates, a total of 853 references remained. Through other sources 1532 additional records were identified leading to a total number of 2385 screened records.

Out of those, 403 were identified and selected for full text evaluation, which led to the exclusion of 368 studies. Only 35 articles published between 1987 and 2016 met all inclusion criteria. A list of excluded studies along with the reason for exclusion is available from the authors upon request. The most frequent reason for exclusion was not meeting our age group target.

Among the included studies, there were 22 SR and MA, 11 RCT, and 2 OS. An overview of the main characteristics and quality of the included studies is presented in Tables 2, 3, 4, 5, 6, 7, 8, 9, 10 and 11. PAI were tested for the following indications: ASA in primary [28–35] and secondary prevention of cardiovascular disease (CVD) [36], ASA in the primary and secondary prevention of stroke in patients with and without AF [30, 34, 37–50]ADP-receptor inhibitors in secondary prevention of cardiovascular events [51, 52] and stroke/TIA [52–54], and dipyridamol in secondary prevention of stroke [55, 56]. Regarding the demographics of the sample, the mean age of participants in the included studies ranges between 57 to 84.6 years. This wide range is due to ten [28, 29, 31, 33, 36, 51, 52, 56, 57] studies that were included because of a subgroup analysis of people aged above 65 years despite a mean age below the threshold. Polypharmacy was not assessed in any of our included studies. None of the included papers reported on the outcomes quality of life, hospitalisation and life expectancy. Information on the presence of comorbidities was reported by 26 of the included studies, mostly consisting of the presence of cardiovascular risk factors or cardiovascular diseases such as stroke and TIA. Coincident medications were declared in 7 of 40 included references. Frailty was only reported by one study [58] and cognitive status by none (see Tables 9, 10 and 11).

## Effectiveness and safety of PAI

### ASA in the primary prevention of CVD

Three SR/MA [28–30] and five RCT [31–35] were included, which examined the primary prevention of CVD. Tha MA of Baigent et al. [28] detected an insignificant reduction in the occurrence of a composite endpoint of serious vascular events including myocardial infarction, stroke, or death from a vascular cause (including sudden death, pulmonary embolism, haemorrhage) in the subgroup of participants older than 65 years (RR 0.88; 95% CI: 0.77–1.01). Even in the complete study group, the benefit of ASA with an absolute risk reduction (ARR) of 0.06% per year for serious vascular events was very low (number needed to treat (NNT) = 1666 per year). Moreover, there was no difference in vascular or all cause mortality between the ASA group and the placebo group (0.19% vs. 0.19% per year; *p* = 0.7), whereas the risk of major gastrointestinal and extracranial bleeds increased under a treatment with ASA (0.10% vs. 0.07% per year, *p* ≤ 0.0001) (secondary endpoints and adverse effects not calculated for older subgroup).

The study of Kjeldsen et al. (2000) with 18,790 participants [31] revealed a significant relative risk reduction in the occurrence of myocardial infarction in the subgroup of participants ≥65 years (RR 0.62; 95% CI: 0.38–0.98; *p* = 0.04), but the relative risk of major cardiovascular events was not significantly reduced in the age group ≥65 years (RR 0.92; 95% CI: 0.74–1.15; *p* = 0.47).

The clinical trial of Ikeda et al. (2014) [35] including 14,464 adults with a mean age of 70 years analysed the impact of ASA on the risk of cardiovascular events in older Japanese patients with multiple atherosclerotic risk factors in comparison to placebo. The primary endpoint of this study was a composite of death from cardiovascular causes (myocardial infarction, stroke, and other cardiovascular causes), nonfatal stroke (ischemic or haemorrhagic), and nonfatal myocardial infarction. Overall, no significant difference in the occurrence of the composite endpoint was observed between the two groups (hazard ratio (HR): 0.94; 95% CI: 0.77–1.15; *p* = 0.54). Moreover, in comparison to the placebo group, the treatment with ASA was associated with a significant increased risk of extracranial haemorrhage requiring transfusion or hospitalization (HR for ASA: 1.85 (95% CI: 1.22–2.81); *p* = 0.004), absolute risk increase 0.35, number needed to harm 286).

Concerning the risk of bleeding in the primary prevention with ASA in older people, the RCT of Silagy et al. [32] with the oldest participants in this subject area (participants *n* = 400, mean age of participants 73 years) identified more gastrointestinal bleeding events in the ASA group in comparison to the placebo group (3% vs. 0%) with a significant decrease in mean hemoglobin levels (0.33 g/dl vs. 0.11 g/dl; *p* < 0.05). He et al. (1998) [29] conducted in a MA a subgroup-analysis of participants above and below 64 years with regard to the risk of haemorrhagic stroke with ASA in comparison to placebo. In the subgroup of participants ≥64 years, the absolute risk difference between the ASA and placebo group was 34 per 10,000 persons (95% CI: 1–66).

The only benefit for ASA was suggested in the RCT of Ogawa et al. (2008) with 2539 participants [33], (which included only people with diabetes mellitus), where in the subgroup of people older than 65 years the benefit in reducing atherosclerotic events (including fatal or nonfatal ischemic heart disease, fatal or nonfatal stroke, and peripheral arterial disease) was significant (HR 0.68; 95% CI: 0.46–0.99; *p* = 0.047).

### ASA in the secondary prevention of CVD

The trial of Huyhn et al. (2001) with 135 participants [36], analysed the effectiveness of ASA, warfarin, and the combination therapy of ASA and warfarin in participants with prior bypass surgery for the secondary prevention of coronary events. The primary endpoint of this study was a composite of any-cause death, myocardial infarction, or unstable angina requiring a new hospitalization. Monotherapy with ASA as well as ASA plus warfarin were associated with the lowest event rate of the composite endpoint (14.6% warfarin, 11.5% ASA, 11.4% ASA warfarin, *p* = 0.76). For patients aged >65 years, an overall higher event rate was detected, but ASA monotherapy revealed the lowest event rate (41.7, 34.8% and 36.8 events respectively). HR were not reported in this publication.

### ASA in the primary prevention of stroke

Patients with AF: Six SR/MA [38–40, 42, 44, 49] showed conflicting evidence regarding the benefit of ASA compared to placebo in the primary prevention of stroke and all cause mortality. While four of the SR demonstrated no benefit for ASA [38, 40, 44, 49] (OR for stroke 0.70 (95% CI:0.47–1.07) [49], 0.56 (95% CI:0.19–1.65) [38] and RR 0.81 (95% CI:0.65–1.01) [40] and 1.30 (95% CI:0.96–1.72) [44] respectively, and for mortality 0.75 (95% CI:0.54–1.04) [49], 0.87 (95% CI: 0.68–1.12) [38] and RR 0.86 (95% CI:0.69–1.07) [40] and 1.28 (95% CI:0.98–1.65) [44], respectively), two SR [39, 42] found a significant benefit for stroke, but not for mortality (RR of 0.64 (95% CI: 0.44–0.88) for stroke, no data for mortality [42], RR 0.78 (95% CI: 0.62–0.98) for stroke and 0.84 (95% CI:0.67–1.05) for mortality [39]).

In all included trials with ASA [38, 40, 42, 44, 49, 59] except for one MA [39], ASA increased the risk of bleeding, especially for gastrointestinal bleeding in comparison to placebo. In the SR of Coleman et al. (2012) [60], the risk of major gastrointestinal bleeding was three times greater under a treatment with ASA compared with placebo. However, these results did not reach statistical significance (odds ratio (OR) 3.23; 95% CI: 0.56–18.66). Connolly et al. (2013) [61] also showed an increased risk of a subdural haematoma under a treatment with ASA in comparison to placebo (OR 2.2; 95% CI: 0.6–7.8; *p* = 0.6), but this was not significant.

Eleven SR/MA [30, 37–46], one RCT [47] and one OS [48] reported that ASA was less effective in preventing stroke in patients with AF than warfarin. The risk of nonfatal ischemic stroke and systemic embolism was significantly higher with a treatment with ASA compared to warfarin [30, 37, 39, 41, 43, 45, 47, 62]. Apart from the results of the MA of Dogliotti et al. [41], there was no significant difference in mortality between the two groups [38–40, 44, 45, 47]. With the exception of six trials [30, 42, 45–47, 57], bleeding events were significantly less frequent in all included studies [37–41, 43, 44, 48, 60] when patients were treated with ASA compared to warfarin. Concerning the use of new oral anticoagulants (NOAC) in older people, ASA were associated with a higher risk of stroke or systemic embolism than NOACs in all included studies [41, 44, 46, 63]. The MA of Lin et al. (2015) [63] showed that in ≥75 years old people ASA was less beneficial concerning the prevention of stroke and systemic embolism compared to the dabigatran treated group (dabigatran 110 mg vs. ASA rate ratio: 1.31 (95% CI: 0.84–2.07). Concerning the risk of bleeding inconsistent results were detected. ASA was associated with a decreased risk of bleeding events in comparison to NOACs (apixaban vs. ASA: OR 0.88; 95% CI: 0.31–2.18 [41]; ASA vs. edoxaban: RR 2.41; 95% CI: 1.02–6.80) [44]. However, in the SR of Cameron et al. (2014) [46], ASA increased the risk of major bleeding events (ASA <100 mg/d vs. edoxaban 30 mg/d: OR 2.27; 95% CI: 1.26–4.1).

### Patients without AF

The clinical trial of Uchiyama et al. [50] with 14,464 participants (mean age 70 years) analysed the impact of ASA on the risk of stroke and intracranial haemorrhage in older Japanese patients without AF in comparison to placebo. Overall, no significant difference in the occurrence of the cumulative rate of fatal or nonfatal stroke was observed between the two groups (HR: 0.92; 95% CI: 0.74–1.16; *p* = 0.51). Five years after randomization, the cumulative rate of fatal or nonfatal stroke in the ASA group was 2.068% (95% CI: 1.75–2.44) as opposed to 2.29% (95% CI: 1.96–2.69) in the placebo group (HR 0.927; 95% CI: 0.741–1.160; *p* = 0.509). Moreover, in comparison to the placebo group, a non-significant reduction of the risk of ischemic stroke or transient ischemic attack was observed in the ASA group (HR 0.783; 95% CI: 0.606–1.012; *p* = 0.061). A treatment with ASA, was associated with a non-significant increase in risk of intracranial haemorrhage in comparison to the placebo group (HR 1.46; 95% CI: 0.956–2.237; *p* = 0.078).

## ASA in the secondary prevention of stroke

### Patients with AF

In these patients, ASA in comparison to placebo showed a higher reduction in secondary than in primary prevention [39, 40]. The ARR of stroke was between 1.5% (NNT = 67) [39] and 0.8% per year (NNT= 125) [40] in the primary prevention trials, and 2.5% per year (NNT = 40) in the secondary prevention trials [39, 40]. In the EAFT trial with 1007 participants [34] no significant reduction in the risk of a recurrent stroke by ASA in comparison to placebo was observed (HR 0.86; 95% CI: 0.64–1.15), while the risk of bleeding non-significantly increased under the treatment with ASA (HR 1.3; 95% CI: 0.8–2.15). Warfarin was much more effective than ASA in the secondary prevention of stroke leading to a significant relative risk reduction of 40% in the occurrence of a recurrent stroke (HR 0.60; 95% CI: 0.41–0.87; *p* = 0.008) [34]. On the other hand, the risk of bleeding was 2.8 fold higher in the warfarin group than in the ASA group (HR 2.8; 95% CI: 1.7–4.8; *p* < 0.001) [34].

### Patients without AF

One study [58] including 505 patients with cerebral infarction, minor or major stroke and a mean age of 68 years analysed the secondary prevention of stroke with ASA in comparison to placebo. The primary endpoints of this study were the recurrence of stroke and death. The incidence of stroke recurrence was 6.3% in the ASA treated group and 6.4% in those randomised to placebo. The OR for stroke recurrence and death comparing ASA to placebo was 1.04 (95% CI: 0.68–1.58), reflecting no significant difference between both groups.

### ADP-receptor inhibitors in the secondary prevention of CVD

One RCT including 13,608 adults with a mean age of 61 years and a subgroup analysis with people older than 75 years, compared prasugrel and clopidogrel for the management of acute coronary syndromes with scheduled percutaneous coronary intervention [51]. The primary endpoint of this study was a composite of cardiovascular mortality, non-fatal myocardial infarction, or non-fatal stroke. In all included patients, the composite primary endpoint (as mentioned above) was reached in 12.1% of patients randomised to clopidogrel and 9.9% of those randomised to prasugrel (HR 0.81; 95% CI: 0.73–0.90; *p* ≤ 0.001). Moreover, prasugrel was more effective in reducing the rates of myocardial infarction (9.7% for clopidogrel vs. 7.4% for prasugrel; *p* ≤ 0.001), urgent target-vessel revascularization (3.7% vs. 2.5%; *p* ≤ 0.001), and stent thrombosis (2.4% vs. 1.1%; *p* ≤ 0.001). Several subgroup-analyses were carried out. One subgroup-analysis of participants aged ≥75 years considered the composite endpoint of death from any cause, nonfatal myocardial infarction, nonfatal stroke, or non-CABG-related nonfatal major bleeding. It showed that in the subgroup of patients older than 75 years, there was no benefit of prasugrel in comparison to clopidogrel regarding this composite endpoint (HR 0.99; 95% CI: 0.81–1.21; *p* = 0.92). Another subgroup-analysis examined the combined endpoint of death from any cause, nonfatal myocardial infarction, and nonfatal stroke under a treatment with prasugrel or clopidogrel in three different age groups (<65 years, 65–74 years, and ≥75 years). In the age group of patients <65 the combined endpoint (as mentioned above) was reached in 8.1% in the prasugrel group compared to 10.6% in the clopidogrel group (risk reduction 25%, HR not reported). In the age group between 65 years and 74 years the occurrence of the combined endpoint was 10.7% in the prasugrel group and 12.3% in the clopidogrel group (risk reduction 14%, no HR reported). In the age group of participants ≥75 years, the risk reduction attributed to prasugrel in comparison to clopidogrel was the lowest of the considered three age groups (17.2% prasugrel group, 18.3% clopidogrel group, risk reduction of 6%, HR or OR not reported). The MA of Zhou et al. [52] with 7 trials including 48,248 participants, investigated the risks and benefits of a dual therapy with ASA and clopidogrel vs. monotherapy for the secondary prevention of cardiovascular and cerebrovascular events (see below). The population, included in this MA, were a mixed population. The participants had for example atrial fibrillation, multiple atherothrombotic risk factors, previous coronary artery bypass grafting/PCI or acute coronary syndromes without ST-segment elevation. The combination therapy was non-significantly more effective than the single drug therapy alone in reducing the rate of major cardiovascular events (9% RR reduction; 95% CI: 2–17) when all participants were included. The relative risk of MI was decreased by 14% (RRR 14%; 95% CI: 3–24). Overall, the ARR of major cardiovascular events due to the combination therapy was 1.06 with a NNT of 83. On the other hand, the combination therapy resulted in a significant 62% RR increase of major bleeding events (95% CI: 26–108) when compared to single drug therapy. For the subgroup analysis of participants older than 65 years, a comparison between the combination therapy and a monotherapy with ASA was performed. In the older participants (≥65 years), the reduction of major cardiovascular events was marginally significant (≥65 years RR: 0.90; 95% CI: 0.83–0.98), whereas the risk of major bleeding events under a treatment with ASA plus clopidogrel vs. ASA monotherapy was significantly higher (≥65 years: RR: 1.56; 95% CI: 1.29–1.89).

### ADP- receptor inhibitors in the secondary prevention of stroke and/or transient ischemic attack

The MA of Zhou et al. [52] described above also investigated, the secondary prevention of cardiovascular events, and the secondary prevention of stroke. With regard to this outcome, the greatest reduction was detected in the occurrence of stroke (RR 16%; 95% CI: 1–28).

In the RCT of Diener et al. (2004) with 7599 participants [53] the benefit to risk ratio did not show the additional clinical value of adding ASA to clopidogrel in high-risk patients with transient ischaemic attack or ischaemic stroke. A subgroup analysis (*n* = 4537) by age (≥65 years) showed that the event rate for clopridogrel plus ASA was 17.4% and for clopidogrel plus placebo 17.7%.

The dual antiplatelet therapy (DAPT) with ASA and clopidogrel was associated with an increased risk of 30-day major stroke, spontaneous MI, all-cause mortality, and combined lethal and major bleeding in the DAPT group compared to monotherapy even in patients who underwent Transcatheter Aortic Valve Implantation (TAVI) (OR 1.88; 95% CI: 1.00–3.56). The biggest increase was detected in the occurrence of lethal and major bleeding events (OR 2.62; 95% CI: 1.29–5.33) [54].

### Dipyridamol (DP) in the secondary prevention of stroke

The MA of Leonardi-Bee et al. [55] with 11,459 participants including a subgroup-analysis of participants older than 65 years identified a non-significant decrease in the reoccurrence of stroke under treatment with DP in comparison to placebo (OR 0.82; 95% CI: 0.68–1.00). In the subgroup of participants ≥65 years the reduction of stroke was non-significant (DP vs. placebo subgroup ≥65 years: OR 0.81; 95% CI: 0.65–1.02). The combination therapy of ASA + DP in comparison to an ASA monotherapy revealed a significant reduction of stroke [55, 56] (ASA + DP vs. ASA monotherapy: Age ≥ 65 years: OR 0.78; 95% CI: 0.63–0.97) [55]. There was no difference in mortality between the two treatment groups (ASA + DP vs. ASA: HR 1.01, 95% CI 0.87–1.17) [56].

## Quality appraisal of included studies

### SR and MA

Table 5 displays the results of quality appraisal of the SR and MA. One MA [52] fulfilled all requirements of the AMSTAR appraisal tool. Several quality deficits were detected when evaluating the other studies using the AMSTAR appraisal tool. In all included MA/SR an a priori design was provided. A duplicate study selection and data extraction were missing in the MA/SR of Baigent et al. [28], Lip et al. [45] and Cooper et al. [42]. In the SR/MA of Leonardi-Bee et al. [55], Taylor et al. [37], and Assiri et al. [44] this information was not available. A comprehensive literature search was not performed in the MA/SR of Connolly et al. [61] and He et al. [29]. Eleven MA [28, 29, 38, 40–45, 56, 60] did not search for grey literature. Quality appraisal of the included studies was not performed in 10 MA/SR [28, 29, 39, 41–43, 45, 46, 56, 61]. Possible conflicts of interest were not declared in four MA [39, 42, 60, 61]. All included SR/MA described the characteristics of the included studies. The likelihood of publication bias was presented in seven MA/SR [37, 43, 45, 52, 54, 60, 63].

### RCTs

Table 6 displays the results of quality appraisal of the RCTs. An appropriate random sequence generation was used in seven [31, 33–35, 50, 53, 58] of 11 RCTs. In four RCTs [32, 36, 47, 51], the random sequence was unclear. Allocation concealment was fulfilled in six studies [33–35, 50, 53, 58] and unclear in five studies [31, 32, 36, 47, 51]. Serious limitations were found in blinding of personnel and participants in five RCTs [33–35, 47, 50], whereas Huynh et al. [36] and Britton et al. [58] performed appropriate blinding of personnel and participants. In four RCTs [31, 32, 51, 53] this remained unclear. The outcomes were unlikely to be influenced by a lack of blinding in five studies [33–35, 50, 58]. The blinding of trials was appropriate in five studies [33–35, 50, 58]. In the remaining studies the blinding outcome was unclear. In the study of Ogawa et al. [33], a high risk for selective reporting was detected due to a missing representation of adverse events in the subgroup analysis of adults ≥65 years of age. Inclusion and exclusion criteria for participants and the primary outcomes were clearly defined and stated in all studies. In two trials [31, 36] differences between the treatment groups after randomisation were identified. The loss to follow-up was less than 5% in six trials [31, 32, 36, 51, 53, 58] whereas the RCT of Ogawa et al. [33], the EAFT trial [34] and the clinical trial of Ikeda et al. [35] had a higher loss to follow-up. In the two other trials [47, 50] it remained unclear. Conflicts of interests were stated in all RCTs except in two trials [31, 34]. In the EAFT [34] trial a high risk for biased selection of participants was detected because all participants who were not eligible for a treatment with warfarin (e.g. due to previous bleeding events) were assigned to the ASA group.

### Observational studies

Table 7 displays the results of quality appraisal of the OS. In the two included OS [48, 59] we could not identify important confounding factors due to a lack of information and an undersized database. The results could be influenced by a lack of blinding of personnel and participants in all included studies.

### Development of recommendations

We developed three recommendations which are presented in Table 8. One recommendation was rated as strong with moderate quality of evidence. The two other recommendations were assessed as weak with low quality of evidence. Table 8 reports on the main articles, which constitute the evidence base for each recommendation, although all included studies were taken into account for the risk/benefit balance during the review process. The quality appraisal of each RCT included in the SR and MA provided the evidence base for the recommendations. All quality appraisals were considered in assessing the quality of the evidence of the recommendations and are available from the authors upon request. Based on the evaluated evidence we formulated three recommendations. The first recommendation deals with the use of ASA in the primary prevention of CVD and stroke in older people without diabetes. The strength of the recommendation is weak and the quality of the evidence was judged as low. Concerning the primary prevention of CVD and stroke in the elderly with ASA, no benefit could be shown in patients without AF compared to placebo. Moreover, the risk of haemorrhagic stroke [28, 29], major gastrointestinal [28, 32] and other extracranial non-fatal bleeds [28, 31, 35] were significantly increased. In contrast, for people with diabetes mellitus the trial of Ogawa et al. [33] showed that the greatest benefit of a treatment with ASA in comparison to placebo was detected in the subgroup of participants older than 65 years. Due to this effect, adults with diabetes mellitus were not included in our recommendation. However, a high risk for selective reporting was detected in this study due to a lack of reporting adverse events in the subgroup analysis of adults ≥65 years of age.

Overall, we reached similar conclusions to the Beers criteria for potentially inappropriate medications in older people, which recommend using ASA with caution in adults older than 80 years for primary prevention of CVD [64, 65].

The second recommendation was developed based on the evidence of MA of Zhou et al. [52] and the RCT of Diener et al. [53]. We recommend avoiding the combination of a dual therapy in the secondary prevention of TIA and stroke with clopidogrel and ASA and to consider monotherapy instead. The evidence shows that a dual therapy increases the risks of bleeding complications and is not beneficial in the secondary prevention of vascular events, especially in the subgroup of adults aged 65 years or older. Adults with another indication for dual therapy (see Table 8) must be excluded from this recommendation. Due to the high quality of the evidence base the strength of the recommendation was rated as strong whereas the quality of evidence was downgraded to moderate quality caused by the indirectness of results. This recommendation was similar to the recommendation of the STOPP/START criteria for potentially inappropriate prescribing in older people, which recommends to stop a dual therapy with ASA and clopidogrel for secondary prevention of stroke (expections are: the patient underwent coronary stenting in the previous 12 months or has a high grade symptomatic carotid arterial stenosis) [66].

The third recommendation is to discontinue the use of ASA for the primary prevention of stroke in older adults with AF (including adults older than 75 years), because current evidence points at an unfavourable risk/benefit ratio for ASA compared to placebo. None of the identified SR demonstrated a benefit regarding mortality, and only two older SR [39, 42] appear to show a benefit regarding stroke. The most recent and reliable SR including a comprehensive network meta-analysis does not show this benefit [44]. Instead, the use of a Vitamin K Antagonist should be considered. This recommendation is based on the evidence of eleven SR/MA [30, 37–41, 46, 57, 63] and one clinical trial [47]. The recommendation was rated as weak and the quality of the evidence as low. The evidence showed a superiority of warfarin in the prevention of cerebrovascular diseases. In regard to the risk of bleeding, contrasting results were found. With the exception of three trials [46, 47, 57], bleeding events were significantly more frequent when compared to ASA. However, the trial [47] with the oldest participants suggested a benefit of warfarin over ASA in octogenarians. There were significantly more ischemic strokes and systemic embolism with ASA than with warfarin but there were significantly fewer adverse events (including bleeding) with warfarin than ASA, assuming a safe handling even in adults older than 80 years. The dose of ASA and the target-INR in the included studies were roughly comparable. A major limitation of this recommendation is that the dose of ASA of 300 mg per day (as it was used in several studies) was higher than the usual applied dose.

We were limited to providing three recommendations to stop treatments because of a lack of reliable studies in our age group. In relation to secondary prevention, the recommendations for the use of PAI are mainly based on studies of younger patients, and it is currently unknown whether these recommendations are transferable to older people. Nonetheless, taking current best evidence regarding younger patients into account, it does not seem justified to formulate a stop recommendation for older people.

## Discussion

Our systematic review examined the benefits and risks of the treatment with PAI for the management of cardiovascular, cerebrovascular and peripheral vascular diseases in older people. This systematic review is part of a compilation of systematic reviews on commonly used drugs in older people and aimed to identify the evidence to develop recommendations on when to discontinue the inappropriate use of these medications in older adults. Based on the evaluated evidence we formulated three recommendations.

Our SR has strengths as well as limitations. To the best of our knowledge, this is the first SR that has searched the evidence on the use of PAI specifically amongst older people. We followed a standard methodology as recommended by the Cochrane collaboration and the PRISMA statement, used a predefined step-wise search approach and piloted our search strategy. This systematic search strategy has the advantage that the search strategy is transparent and reproducible and will have utility in assessing the evidence for treatments aimed specifically at older people. Unfortunately, many papers had to be excluded as they did not report on the evidence for treatments in older people reflecting the lack of studies in this age group. An example was the lack of evidence concerning the secondary prevention of stroke and cardiovascular disease with ASA in comparison to placebo. The only study for secondary prevention of CVD, that met our inclusion criteria, was a small study with 132 participants and insignificant results. Current guidelines on recommendations to prescribe ASA in the secondary prevention of CVD and stroke are therefore based on study evidence derived from younger patients [67]. We do not know whether the benefits shown in these studies are also applicable to older people. There are ongoing studies targeted at filling this evidence gap. The largest of these studies is the ASPREE Trial (study protocol published 2013) [68], taking place in Australia. It included 16,700 participants aged 70 years and older and aims to analyse the impact of daily low-dose aspirin on cardiovascular disease (heart attack and stroke) in older people. The results of the ASPREE trial are expected to be published in 2018.

Another limitation is that our search strategy resulted in SR and MA with overlapping studies (see additional file 2). Altogether, 143 studies were included in the SR and MA and out of these studies, 40 studies were counted repeatedly. This probably meant that outcomes from these overlapping studies would be weighted more positively in our analyses compared to studies, which have only been included once. Despite the overlap, we decided to include all SR and MA because they offered additional relevant information.

We included two different types of MA namely standard MA based on head-to-head comparisons and network MA making indirect comparisons. Although the strength of evidence of network MA in general is considered to be weaker than that of standard MA, this would not have led a different conclusion in our SR. It is important to note that due to our methodology we could not take into account the strength of evidence of network MA which is generally weaker.

During the development of our recommendations we weighted the benefits and risks for using platelet aggregation inhibitors in older people [19]. We did not assess the material for people of other ages and we did not look for possible start recommendations because our study had the specific aim of helping to reduce polypharmacy in older people. The widespread use of ASA contributes significantly to the problem of polypharmacy. [69]. With the implementation of our recommendations we hope to contribute to a reduction in the treatment with PAI and hence reduce inappropriate polypharmacy. We hope our recommendations will lead to the development of new guidelines specifically addressing the drug treatment of old and multi-morbid adults. We are currently using these recommendations in an electronic decision support tool aimed at reduce polypharmacy in a multicenter, randomised, controlled PRIMA-eDS trial with 3900 patients [18].

## Conclusions

Based on the evaluated evidence, this systematic review was able to develop three recommendations. The use of ASA for the primary prevention of CVD and the combination therapy of ASA and clopidogrel for the secondary prevention of vascular events in older people may not be justified when the risk-benefit ratio is taken into account. The use of warfarin instead of ASA in older patients with AF may be recommended. To improve the effectiveness and reduce the risks of stroke prevention therapy in older people with AF, the discontinuation of ASA for the primary prevention of stroke should be considered and oral anticoagulants could be used instead (low quality of evidence).

Older patients with multimorbidity and polypharmacy are underrepresented in clinical trials. None of the articles, that we identified, reported on patients with polypharmacy. We were therefore not able to develop recommendations for reducing polypharmacy in patients who are old and with multi-morbidity. The benefits of many treatments for these patient groups are less clear and further good quality studies are needed for example RCTs investigating the individualised assessment of multi-morbid people with polypharmacy (including PAI).

We expect our recommendations in addition with the other recommendations of the PRIMA-eDS trial to contribute to the development of new guidelines specifically addressing the drug treatment of old adults with multi-morbidity.

## Additional files

Additional file 1: Search string search 1 and 2. (DOCX 102 kb)

Additional file 2: Overlapping studies. (DOCX 211 kb)

## Abbreviations

AF: Atrial fibrillation
AMSTAR: Assessment of multiple systematic reviews
ARR: Absolute risk reduction
ASA: Acetylsalicylic acid
CASP: Critical Appraisal Skills Programme
CI: Confidence interval
CVD: Cardiovascular disease
DAPT: Dual- antiplatelet therapy
DARE: Database of Abstracts or Reviews of Effects
DP: Dipyridamole
GRADE: Grading of Recommendations Assessment, Development and Evaluation
HR: Hazard ratio
HTA: Health Technology Assessment
IPA: International Pharmaceutical Abstracts
MA: Meta-analysis
NNT: Number needed to treat
OR: Odds ratio
OS: Observational study
PAI: Platelet aggregation inhibitors
PICOS: Population, intervention, comparison, outcomes and study design
PRIMA-eDS: Polypharmacy in chronic diseases: Reduction of Inappropriate Medication and Adverse drug events in older populations by electronic Decision Support
RCT: Randomised controlled trial
SR: Systematic review
TIA: Transient ischaemic attack
VKA: Vitamin K Antagonist

## References

[1] Hovstadius B (2010). Increasing polypharmacy - an individual-based study of the Swedish population 2005-2008. BMC Clin Pharmacol.

[2] Onder G, et al. Advanced age and medication prescription: More years, less medications? A nationwide report from the Italian medicines agency. J Am Med Director Assoc. 2016;17(2):168-72. 10.1016/j.jamda.2015.08.009 26441359

[3] Flaherty JH (2000). Polypharmacy and hospitalization among older home care patients. J Gerontol A Biol Sci Med Sci.

[4] Gregg D, Goldschmidt-Clermont PJ (2003). Platelets and cardiovascular disease. Circulation.

[5] Silber S (2010). Evidence-based management of ST-segment elevation myocardial infarction (STEMI): latest guidelines of the European Society of Cardiology (ESC) 2010. [German] Evidenzbasiertes Vorgehen beim ST-Strecken-Hebungsinfarkt (STEMI): Neueste Leitlinien der Europaischen Gesellschaft fur Kardiologie (ESC) 2010. Herz.

[6] Perk J (2013). European guidelines on cardiovascular disease prevention in clinical practice (version 2012). The fifth joint task force of the European Society of Cardiology and other societies on cardiovascular disease prevention in clinical practice (constituted by representatives of nine societies and by invited experts). G Ital Cardiol (Rome).

[7] Lüllmann H, Mohr K, Hein L. Pharmakologie und Toxikologie. Georg Thieme Verlag. 2010;207(9):314.

[8] Patrono C (2005). Low-dose aspirin for the prevention of atherothrombosis. N Engl J Med.

[9] Howard RL (2007). Which drugs cause preventable admissions to hospital? A systematic review. Br J Clin Pharmacol.

[10] Kongkaew C (2013). Risk factors for hospital admissions associated with adverse drug events. Pharmacotherapy.

[11] Davies EC (2010). Emergency re-admissions to hospital due to adverse drug reactions within 1 year of the index admission. Br J Clin Pharmacol.

[12] Salvi F (2012). Adverse drug events as a cause of hospitalization in older adults. Drug Saf.

[13] Fick DM (2003). Updating the beers criteria for potentially inappropriate medication use in older adults: results of a US consensus panel of experts. Arch Intern Med.

[14] Sorensen HT (2000). Risk of upper gastrointestinal bleeding associated with use of low-dose aspirin. Am J Gastroenterol.

[15] Franchini M (2006). Hemostasis and aging. Crit Rev Oncol Hematol.

[16] Van Spall HG (2007). Eligibility criteria of randomized controlled trials published in high-impact general medical journals: a systematic sampling review. JAMA.

[17] Boyd CM (2005). Clinical practice guidelines and quality of care for older patients with multiple comorbid diseases: implications for pay for performance. JAMA.

[18] Sonnichsen A (2016). Polypharmacy in chronic diseases-reduction of inappropriate medication and adverse drug events in older populations by electronic decision support (PRIMA-eDS): study protocol for a randomized controlled trial. Trials.

[19] Martinez YM, R.-G.A., Reeves D, Ediriweera de Silva RE, Esmail A, Kunnamo I, Rieckert A, Sommerauer C, Sönnichsen A, A set of systematic reviews to help reduce inappropriate prescribing to older people: study protocol*.* BMC Geriatrics. 2017;17(1). 10.1186/s12877-017-0570-9 PMC5647557 29047332

[20] Shea BJ (2007). Development of AMSTAR: a measurement tool to assess the methodological quality of systematic reviews. BMC Med Res Methodol.

[21] Shea BJ (2009). AMSTAR is a reliable and valid measurement tool to assess the methodological quality of systematic reviews. J Clin Epidemiol.

[22] Higgins JPT, G.S., (editors). Cochrane handbook for systematic reviews of interventions. Version 5.1.0 [updated March 2011] The Cochrane Collaboration, 2011. Available from http://handbook-5-1.cochrane.org/. 2011. Last accessed 18 Aug 2017.

[23] Critical Appraisal Skills Programme. 11 questions to help you make sense of case control study. 2013; Available from: https://hhs.hud.ac.uk/lqsu/Useful/critap/Case%20Control%20Study%20Checklist/CASP-Case-Control-Study-Checklist-31.05.13.pdf. Accessed 18 Aug 2018.

[24] Critical Appraisal Skills Programme. 12 questions to help you make sense of cohort study. 2013; Available from: https://hhs.hud.ac.uk/lqsu/Useful/critap/Cohort%20Study%20Checklist/CASPCohort-Study-Checklist-31.05.13.pdf. Accessed 18 Aug 2017.

[25] Guyatt GH (2008). Going from evidence to recommendations. BMJ.

[26] Guyatt GH (2008). What is “quality of evidence” and why is it important to clinicians?. BMJ.

[27] Guyatt GH (2008). GRADE: an emerging consensus on rating quality of evidence and strength of recommendations. BMJ.

[28] Baigent C (2009). Aspirin in the primary and secondary prevention of vascular disease: collaborative meta-analysis of individual participant data from randomised trials. Lancet.

[29] He J (1998). Aspirin and risk of hemorrhagic stroke: a meta-analysis of randomized controlled trials. JAMA.

[30] Aguilar MI, Hart R, Pearce LA. Oral anticoagulants versus antiplatelet therapy for preventing stroke in patients with non-valvular atrial fibrillation and no history of stroke or transient ischemic attacks. Cochrane Database Syst Rev. 2007;(3):Cd006186. 10.1002/14651858.CD006186.pub2 PMC13591242 17636831

[31] Kjeldsen SE (2000). Influence of gender and age on preventing cardiovascular disease by antihypertensive treatment and acetylsalicylic acid. The HOT study. Hypertension optimal treatment. J Hypertens.

[32] Silagy CA (1993). Adverse effects of low-dose aspirin in a healthy elderly population. Clin Pharmacol Ther.

[33] Ogawa H (2008). Low-dose aspirin for primary prevention of atherosclerotic events in patients with type 2 diabetes: a randomized controlled trial. JAMA.

[34] Group, E.A.F.T.S (1993). Secondary prevention in non-rheumatic atrial fibrillation after transient ischaemic attack or minor stroke. EAFT (European Atrial fibrillation trial) study group. Lancet.

[35] Ikeda Y (2014). Low-dose aspirin for primary prevention of cardiovascular events in Japanese patients 60 years or older with atherosclerotic risk factors: a randomized clinical trial. JAMA.

[36] Huynh T (2001). Aspirin, warfarin, or the combination for secondary prevention of coronary events in patients with acute coronary syndromes and prior coronary artery bypass surgery. Circulation.

[37] Taylor FC, Cohen H, Ebrahim S (2001). Systematic review of long term anticoagulation or antiplatelet treatment in patients with non-rheumatic atrial fibrillation. BMJ.

[38] Segal JB (2000). Prevention of thromboembolism in atrial fibrillation: a meta-analysis of trials of anticoagulants and antiplatelet drugs. J Gen Intern Med.

[39] Hart RG (1999). Antithrombotic therapy to prevent stroke in patients with atrial fibrillation: a meta-analysis. Ann Intern Med.

[40] Hart RG, Pearce LA, Aguilar MI (2007). Meta-analysis: antithrombotic therapy to prevent stroke in patients who have nonvalvular atrial fibrillation. Ann Intern Med.

[41] Dogliotti A, Paolasso E, Giugliano RP (2014). Current and new oral antithrombotics in non-valvular atrial fibrillation: a network meta-analysis of 79 808 patients. Heart.

[42] Cooper NJ (2006). Mixed comparison of stroke prevention treatments in individuals with nonrheumatic atrial fibrillation. Arch Intern Med.

[43] Andersen LV (2008). Warfarin for the prevention of systemic embolism in patients with non-valvular atrial fibrillation: a meta-analysis. Heart.

[44] Assiri A (2013). Mixed treatment comparison meta-analysis of aspirin, warfarin, and new anticoagulants for stroke prevention in patients with nonvalvular atrial fibrillation. Clin Ther.

[45] Lip GY, Edwards SJ (2006). Stroke prevention with aspirin, warfarin and ximelagatran in patients with non-valvular atrial fibrillation: a systematic review and meta-analysis. Thromb Res.

[46] Cameron C (2014). Systematic review and network meta-analysis comparing antithrombotic agents for the prevention of stroke and major bleeding in patients with atrial fibrillation. BMJ Open.

[47] Liu X (2014). Warfarin compared with aspirin for older Chinese patients with stable coronary heart diseases and atrial fibrillation complications. Int J Clin Pharmacol Ther.

[48] Burton C (2006). The safety and adequacy of antithrombotic therapy for atrial fibrillation: a regional cohort study. Br J Gen Pract.

[49] Aguilar MI, Hart R (2005). Antiplatelet therapy for preventing stroke in patients with non-valvular atrial fibrillation and no previous history of stroke or transient ischemic attacks. Cochrane Database Syst Rev.

[50] Uchiyama S (2016). Aspirin for stroke prevention in elderly patients with vascular risk factors: Japanese primary prevention project. Stroke.

[51] Wiviott SD (2007). Prasugrel versus clopidogrel in patients with acute coronary syndromes. N Engl J Med.

[52] Zhou YH (2012). Effects of combined aspirin and clopidogrel therapy on cardiovascular outcomes: a systematic review and meta-analysis. PLoS One.

[53] Diener HC (2004). Aspirin and clopidogrel compared with clopidogrel alone after recent ischaemic stroke or transient ischaemic attack in high-risk patients (MATCH): randomised, double-blind, placebo-controlled trial. Lancet.

[54] Gandhi S (2015). Comparison of dual-antiplatelet therapy to mono-antiplatelet therapy after Transcatheter aortic valve implantation: systematic review and meta-analysis. Can J Cardiol.

[55] Leonardi-Bee J (2005). Dipyridamole for preventing recurrent ischemic stroke and other vascular events: a meta-analysis of individual patient data from randomized controlled trials. Stroke.

[56] Halkes PH (2008). Dipyridamole plus aspirin versus aspirin alone in secondary prevention after TIA or stroke: a meta-analysis by risk. J Neurol Neurosurg Psychiatry.

[57] Warkentin AE (2012). Bleeding risk in randomized controlled trials comparing warfarin and aspirin: a systematic review and meta-analysis. J Thromb Haemost.

[58] Britton M, Helmers C, Samuelsson K (1987). High-dose acetylsalicylic acid after cerebral infarction*.* A Swedish cooperative study. Stroke.

[59] Sam C (2004). Warfarin and aspirin use and the predictors of major bleeding complications in atrial fibrillation (the Framingham heart study). Am J Cardiol.

[60] Coleman CI (2012). Effect of pharmacological therapies for stroke prevention on major gastrointestinal bleeding in patients with atrial fibrillation. Int J Clin Pract.

[61] Connolly BJ (2013). Aspirin therapy and risk of subdural hematoma: meta-analysis of randomized clinical trials. J Stroke Cerebrovasc Dis.

[62] Stroke prevention in atrial fibrillation investigators. Warfarin versus aspirin for prevention of thromboembolism in atrial fibrillation: Stroke Prevention in Atrial Fibrillation II Study. Lancet. 1994;343(8899):687–91. 7907677

[63] Lin L (2015). Clinical and safety outcomes of oral Antithrombotics for stroke prevention in Atrial fibrillation: a systematic review and network meta-analysis. J Am Med Dir Assoc.

[64] The American geriatrics society 2015 beers criteria update expert panel. American geriatrics society 2015 updated beers criteria for potentially inappropriate medication use in older adults. J Am Geriatr Soc. 2015;63(11):2227-46. 10.1111/jgs.13702 26446832

[65] American geriatrics society 2012 beers criteria update expert panel. American geriatrics society updated beers criteria for potentially inappropriate medication use in older adults. J Am Geriatr Soc. 2012;60(4):616–31. 10.1111/j.1532-5415.2012.03923.x PMC3571677 22376048

[66] O'Mahony D (2015). STOPP/START criteria for potentially inappropriate prescribing in older people: version 2. Age Ageing.

[67] Piepoli MF (2016). 2016 European guidelines on cardiovascular disease prevention in clinical practice: the sixth joint task force of the European Society of Cardiology and Other Societies on cardiovascular disease prevention in clinical practice (constituted by representatives of 10 societies and by invited experts) developed with the special contribution of the European Association for Cardiovascular Prevention & rehabilitation (EACPR). Atherosclerosis.

[68] ASPREE Investigator Group. Study design of ASPirin in Reducing Events in the Elderly (ASPREE): a randomized, controlled trial. Contemp Clin Trials. 2013;36(2)555–64. 10.1016/j.cct.2013.09.014 PMC3919683 24113028

[69] Slabaugh SL (2010). Prevalence and risk of polypharmacy among the elderly in an outpatient setting: a retrospective cohort study in the Emilia-Romagna region, Italy. Drugs Aging.

