# Supplementary material for: Effectiveness and patient safety of platelet aggregation inhibitors in the prevention of cardiovascular disease and ischemic stroke in older adults – a systematic review
Source: BMC Geriatr. 2017 Oct 16;17(Suppl 1):225. doi: 10.1186/s12877-017-0572-7 (PMC5647552; doi:10.1186/s12877-017-0572-7)
Supplement: Supplementary file 1 — Search string search 1 and 2. (DOCX 102 kb) [file 12877_2017_572_MOESM1_ESM.docx]

**Search string search 1 and 2: Effectiveness and Patient safety of platelet aggregation inhibitors in the management of cerebrovascular, peripheral artery occlusive and coronary disease in older adults**

**Databases:**

**EBM Reviews - Cochrane Database of Systematic Reviews [search 1]**

**EBM Reviews - Database of Abstracts of Reviews of Effects [search 1]**

**Ovid MEDLINE(R) [search 2]**

**Ovid MEDLINE(R) In-Process & Other Non-Indexed Citations [search 2]**

**Embase [search 2]**

**EBM Reviews - Health Technology Assessment [search 2]**

**International Pharmaceutical Abstracts [search 2]**

| **[# ▲](http://ovidsp.uk.ovid.com/sp-3.8.1a/ovidweb.cgi?&S=DCDLPDGALHHFOEDMFNOKEBEGNLKJAA00&Sort+Sets=descending)** | **Searches** |
| --- | --- |
| **Population** | |
|  | geriatrics.mp. or exp geriatrics/ |
|  | geriatric patient.mp. |
|  | geriatric*.mp. |
|  | (elder$ or geriatric$).ab,ti. |
|  | elder*.mp. |
|  | frail elderly.mp. or exp frail elderly/ |
|  | aged.mp. or exp Aged/ |
|  | old*.mp. |
|  | old* adult*.mp. |
|  | old* people*.mp. |
|  | >65.mp. |
|  | over 65.mp. |
|  | or/1-12 |
| **Condition** | |
|  | exp Stroke/ |
|  | stroke.mp. |
|  | Ischemic Attack, transient.mp. |
|  | exp transient ischemic attack/ |
|  | exp ischemic heart disease/ |
|  | exp peripheral occlusive artery disease/ |
|  | Peripheral Arterial Disease.mp. |
|  | Angina pectoris.mp. |
|  | exp angina pectoris/ |
|  | Atrial fibrillation.mp. |
|  | exp cardiovascular risk/ |
|  | cardiovascular risk.mp. |
|  | exp cerebrovascular accident/ |
|  | exp carotid artery obstruction/ |
|  | exp vascular disease/ |
|  | exp heart muscle ischemia/ |
|  | exp coronary artery disease/ |
|  | exp transluminal coronary angioplasty/ |
|  | exp heart atrium fibrillation/ |
|  | Diabetes Mellitus, Type 2.mp. |
|  | myocardial ischaemia.mp. |
|  | exp Myocardial Infarction/ |
|  | myocardial infarction.mp. |
|  | exp heart infarction/ |
|  | exp heart muscle ischemia/ |
|  | coronary disease.mp. |
|  | angioplasty, balloon, coronary.mp. |
|  | Cardiovascular Diseases.mp. |
|  | exp cardiovascular disease/ |
|  | intermittant claudication.mp. |
|  | or/14-43 |
| **Intervention** | |
|  | exp Platelet Aggregation Inhibitors/ |
|  | Platelet aggregation inhibitors.mp. |
|  | ditazole.mp. |
|  | exp ditazole/ |
|  | cloricromen.mp. |
|  | exp cloricromen/ |
|  | picotamide.mp. |
|  | exp picotamide/ |
|  | exp Ticlopidine/ |
|  | ticlopidine.mp. |
|  | clopidogrel.mp. |
|  | exp clopidogrel/ |
|  | exp acetylsalicylic acid plus clopidogrel/ |
|  | acetylsalicylic acid.mp. |
|  | exp Aspirin/ |
|  | dipyridamole.mp. |
|  | exp dipyridamole/ |
|  | exp acetylsalicylic acid plus dipyridamole/ |
|  | carbasalate calcium.mp. |
|  | exp carbasalate calcium/ |
|  | epoprostenol.mp. |
|  | exp prostacyclin/ |
|  | indobufen.mp. |
|  | exp indobufen/ |
|  | iloprost.mp. |
|  | exp iloprost/ |
|  | abciximab.mp. |
|  | exp abciximab/ |
|  | aloxiprin.mp. |
|  | exp aloxiprin/ |
|  | eptifibatide.mp. |
|  | exp eptifibatide/ |
|  | tirofiban.mp. |
|  | exp tirofiban/ |
|  | triflusal.mp. |
|  | exp triflusal/ |
|  | beraprost.mp. |
|  | treprostinil.mp. |
|  | prasugrel.mp. |
|  | exp prasugrel/ |
|  | cilostazol.mp. |
|  | ticagrelor.mp. |
|  | exp ticagrelor/ |
|  | or/45-87 |
| **Outcome** | |
|  | mortality.mp. or exp mortality/ |
|  | quality of life.mp. or exp quality of life/ |
|  | QOL.mp |
|  | cardiovascular event.mp |
|  | myocardial infarction.mp |
|  | stroke.mp |
|  | hospitalization.mp. or exp hospitalization/ |
|  | hospitalisation.mp. or exp hospitalisation/ |
|  | life expectancy.mp |
|  | cognitive impairment.mp |
|  | cognitive status.mp. |
|  | functional status.mp. |
|  | functional impairment.mp. |
|  | renal failure.mp |
|  | renal insufficiency.mp. or exp renal insufficiency/ |
|  | adverse drug event.mp |
|  | adverse effects.mp. or exp adverse effects/ |
|  | drug toxicity.mp. or exp drug toxicity/ |
|  | safety.mp |
|  | patient safety.mp. or exp patient safety/ |
|  | falls.mp. |
|  | delirium.mp. or exp delirium/ |
|  | or/89-110 |
| **Limits, Study designs** | |
|  | (systematic review.ti. or meta-analysis.pt. or meta-analysis.ti. or systematic literature review.ti. or (systematic review.ti,ab. and review.pt.) or consensus development conference.pt. or practice guideline.pt. or cochrane database of systematic reviews.jn. or acp journal club.jn. or health technology assessment winchester england.jn. or evidence report technology assessment summary.jn. or drug class reviews.ti.) or (clinical guideline.tw and management.tw) or ((evidence based.ti. or evidence-based medicine.sh. or best practice*.ti. or evidence synthesis.ti,ab.) and (review.pt. or diseases category.mp. or behaviour.sh. and behavior mechanisms.mp. or therapeutics.sh. or evaluation studies.pt. or validation studies.pt. or guideline.pt. or pmcbook.mp.)) or ((systematic.tw or systematically.tw or critical.ti,ab. or (study selection.tw.) or (predetermined.tw or inclusion.tw and criteri*.tw) or exclusion criteri*.tw. or main outcome measures.tw. or standard of care.tw. or standards of care.tw.) and (survey.ti,ab. or surveys.ti,ab. or overview*.tw. or review.ti,ab. or reviews.ti,ab. or search*.tw. or handsearch.tw. or analysis.ti,ab. or critique.ti,ab. or appraisal.tw. or (reduction.tw. and (risk.sh. or risk.tw.) and (death.mp or recurrence.mp))) and (literature.ti,ab. or articles.ti,ab. or publications.ti,ab. or publication.ti,ab. or bibliography.ti,ab. or bibliographies.ti,ab. or published.ti,ab. or unpublished.tw. or citation.tw. or citations.tw. or database.ti,ab. or internet.ti,ab. or textbooks.ti,ab. or references.tw. or scales.tw. or papers.tw. or datasets.tw. or trials.ti,ab. or meta-analy*.tw. or (clinical.ti,ab. and studies.ti,ab.) or treatment outcome.sh. or treatment outcome.tw. or pmcbook.mp.)) not (letter.pt. or newspaper article.pt. or comment.pt.) |
