# Supplementary material for: Effectiveness and patient safety of platelet aggregation inhibitors in the prevention of cardiovascular disease and ischemic stroke in older adults – a systematic review
Source: BMC Geriatr. 2017 Oct 16;17(Suppl 1):225. doi: 10.1186/s12877-017-0572-7 (PMC5647552; doi:10.1186/s12877-017-0572-7)
Supplement: Supplementary file 2 — Overlapping studies. (DOCX 211 kb) [file 12877_2017_572_MOESM2_ESM.docx]

| **Included studies of each SR/MA** | | **SR/MA** | | | | | | | | | | | | | | | | | | | | | |
| --- | --- | --- | --- | --- | --- | --- | --- | --- | --- | --- | --- | --- | --- | --- | --- | --- | --- | --- | --- | --- | --- | --- | --- |
|  | | Aguilar, 2005  [49] 5 | Aguilar 2007  [30] 7 | Andersen 2013  [43?] 15 | Assiri 2008  [43] 21 | Baigent 2009  [28] 6 (primary prevention, 16 secondary prevention) | Cameron 2014  [46] 16 | Cooper 2006  [42] 19 | Coleman 2012  [60] 17 | Connolly 2013 [61] 9 | Dogliotti 2014  [41] 20 | Gandhi 2015  [54] 4 | Halkes 2008  [56] 5 | Hart 2007  [40] 29 | Hart 1999  [39] 6 | He 1998 (JAMA)  [29] 16 | Leonardi-Bee 2005 [55] 7 | Lin 2015 [63] 49 (25 randomized controlled studies, 24 nonrandomized studies) | Lip 2006 [45] 13 | Segal 2000 [38] 11 | Taylor 2001 [37] 6 | Warkentin 2012 [57] 8 | Zhou 2012 [52] 7 |
| AFASAK I  1989 [[70](file:///T:\gesundheit\humanmedizin\allgemeinmedizin\PRIMA-eDS\work%20packages%20(WP%2001-%20WP%2012)\WP_02%20SR_recommend\01-plat%20agr%20inhibitors_all%20indic\8-%20Publication\submission%203\Anne\PAI_main%20manuscript_ohne%20Tabellen.doc#_ENREF_70)] | | x | x | x | x |  |  | x | x |  | x |  |  | x |  | x |  | x | x | x | x |  |  |
| AFASAK II  1998 [[71](file:///T:\gesundheit\humanmedizin\allgemeinmedizin\PRIMA-eDS\work%20packages%20(WP%2001-%20WP%2012)\WP_02%20SR_recommend\01-plat%20agr%20inhibitors_all%20indic\8-%20Publication\submission%203\Anne\PAI_main%20manuscript_ohne%20Tabellen.doc#_ENREF_71)] | |  | x | x |  |  | x | x | x |  | x |  |  | x | x |  |  | x | x | x | x | x |  |
| BAATAF  1990 [[72](file:///T:\gesundheit\humanmedizin\allgemeinmedizin\PRIMA-eDS\work%20packages%20(WP%2001-%20WP%2012)\WP_02%20SR_recommend\01-plat%20agr%20inhibitors_all%20indic\8-%20Publication\submission%203\Anne\PAI_main%20manuscript_ohne%20Tabellen.doc#_ENREF_72)] | | x |  |  |  |  |  | x (included in initial analysis but excluded in sensivity analysis) | x |  | x |  |  | x |  |  |  |  | x | x |  |  |  |
| CAFA  1991 [[73](file:///T:\gesundheit\humanmedizin\allgemeinmedizin\PRIMA-eDS\work%20packages%20(WP%2001-%20WP%2012)\WP_02%20SR_recommend\01-plat%20agr%20inhibitors_all%20indic\8-%20Publication\submission%203\Anne\PAI_main%20manuscript_ohne%20Tabellen.doc#_ENREF_73)] | | x |  | x |  |  | x | x |  |  | x |  |  | x |  |  |  | x | x | x |  |  |  |
| SPAF I  1991[[74](file:///T:\gesundheit\humanmedizin\allgemeinmedizin\PRIMA-eDS\work%20packages%20(WP%2001-%20WP%2012)\WP_02%20SR_recommend\01-plat%20agr%20inhibitors_all%20indic\8-%20Publication\submission%203\Anne\PAI_main%20manuscript_ohne%20Tabellen.doc#_ENREF_74)] | | x |  | x |  |  |  | x |  | x | x |  |  | x |  | x |  | x | x | x |  |  |  |
| SPAF II 1994 [[62](file:///T:\gesundheit\humanmedizin\allgemeinmedizin\PRIMA-eDS\work%20packages%20(WP%2001-%20WP%2012)\WP_02%20SR_recommend\01-plat%20agr%20inhibitors_all%20indic\8-%20Publication\submission%203\Anne\PAI_main%20manuscript_ohne%20Tabellen.doc#_ENREF_62)] | |  | x | x | x |  |  | x | x |  | x |  |  | x |  |  |  | x | x | x | x (<75 y. ; >75 y.) | |  |
| SPAF III 1996 [[75](file:///T:\gesundheit\humanmedizin\allgemeinmedizin\PRIMA-eDS\work%20packages%20(WP%2001-%20WP%2012)\WP_02%20SR_recommend\01-plat%20agr%20inhibitors_all%20indic\8-%20Publication\submission%203\Anne\PAI_main%20manuscript_ohne%20Tabellen.doc#_ENREF_75)] | |  |  | x | x |  |  | x (≤75 y.; ≥75 y.) | x |  |  |  |  | x |  |  |  |  | x | x |  |  |  |
| SIFA 1997 [[76](file:///T:\gesundheit\humanmedizin\allgemeinmedizin\PRIMA-eDS\work%20packages%20(WP%2001-%20WP%2012)\WP_02%20SR_recommend\01-plat%20agr%20inhibitors_all%20indic\8-%20Publication\submission%203\Anne\PAI_main%20manuscript_ohne%20Tabellen.doc#_ENREF_76)] | |  |  | x |  |  |  | x | x |  |  |  |  | x |  |  |  |  |  | x | x |  |  |
| SPINAF  1992 [[77](file:///T:\gesundheit\humanmedizin\allgemeinmedizin\PRIMA-eDS\work%20packages%20(WP%2001-%20WP%2012)\WP_02%20SR_recommend\01-plat%20agr%20inhibitors_all%20indic\8-%20Publication\submission%203\Anne\PAI_main%20manuscript_ohne%20Tabellen.doc#_ENREF_77)] | | x |  |  | x |  |  | x | x |  | x |  |  | x |  |  |  |  | x | x |  |  |  |
| ACTIVE W  2006 [[78](file:///T:\gesundheit\humanmedizin\allgemeinmedizin\PRIMA-eDS\work%20packages%20(WP%2001-%20WP%2012)\WP_02%20SR_recommend\01-plat%20agr%20inhibitors_all%20indic\8-%20Publication\submission%203\Anne\PAI_main%20manuscript_ohne%20Tabellen.doc#_ENREF_78)] | |  | x | x | x |  | x |  |  |  | x |  |  | x |  |  |  | x |  |  |  |  |  |
| ATHENS  2006 [[79](file:///T:\gesundheit\humanmedizin\allgemeinmedizin\PRIMA-eDS\work%20packages%20(WP%2001-%20WP%2012)\WP_02%20SR_recommend\01-plat%20agr%20inhibitors_all%20indic\8-%20Publication\submission%203\Anne\PAI_main%20manuscript_ohne%20Tabellen.doc#_ENREF_79)] | |  | x |  |  |  |  |  |  |  |  |  |  | x |  |  |  |  |  |  |  |  |  |
| NASPEAF  2004 [[80](file:///T:\gesundheit\humanmedizin\allgemeinmedizin\PRIMA-eDS\work%20packages%20(WP%2001-%20WP%2012)\WP_02%20SR_recommend\01-plat%20agr%20inhibitors_all%20indic\8-%20Publication\submission%203\Anne\PAI_main%20manuscript_ohne%20Tabellen.doc#_ENREF_80)] | |  | x |  |  |  |  |  | x |  |  |  |  | x |  |  |  |  |  |  |  |  |  |
| PATAF (I)  1999  [[81](file:///T:\gesundheit\humanmedizin\allgemeinmedizin\PRIMA-eDS\work%20packages%20(WP%2001-%20WP%2012)\WP_02%20SR_recommend\01-plat%20agr%20inhibitors_all%20indic\8-%20Publication\submission%203\Anne\PAI_main%20manuscript_ohne%20Tabellen.doc#_ENREF_81)] | |  | x | x |  |  |  | x | x |  | x |  |  | x |  |  |  | x | x |  | x |  |  |
| PATAF (II) 1999 [80] | |  |  |  |  |  |  | x |  |  |  |  |  |  |  |  |  |  |  |  |  |  |  |
| EAFT 1993 [34] | |  |  | x | x |  |  | x | x |  | x |  |  | x |  | x |  |  | x | x |  |  |  |
| WASPO 2007 [[82](file:///T:\gesundheit\humanmedizin\allgemeinmedizin\PRIMA-eDS\work%20packages%20(WP%2001-%20WP%2012)\WP_02%20SR_recommend\01-plat%20agr%20inhibitors_all%20indic\8-%20Publication\submission%203\Anne\PAI_main%20manuscript_ohne%20Tabellen.doc#_ENREF_82)] | |  |  | x | x |  | x (not included in analysis) |  |  |  | x |  |  | x |  |  |  | x |  |  |  | x |  |
| BAFTA 2007 [[83](file:///T:\gesundheit\humanmedizin\allgemeinmedizin\PRIMA-eDS\work%20packages%20(WP%2001-%20WP%2012)\WP_02%20SR_recommend\01-plat%20agr%20inhibitors_all%20indic\8-%20Publication\submission%203\Anne\PAI_main%20manuscript_ohne%20Tabellen.doc#_ENREF_83)] | |  |  | x | x |  | x |  |  |  | x |  |  |  |  |  |  | x |  |  |  | x |  |
| WASH 2004 [[84](file:///T:\gesundheit\humanmedizin\allgemeinmedizin\PRIMA-eDS\work%20packages%20(WP%2001-%20WP%2012)\WP_02%20SR_recommend\01-plat%20agr%20inhibitors_all%20indic\8-%20Publication\submission%203\Anne\PAI_main%20manuscript_ohne%20Tabellen.doc#_ENREF_84)] | |  |  |  |  |  |  |  |  |  |  |  |  |  |  |  |  |  |  |  |  | x |  |
| HELAS 2006 [[85](file:///T:\gesundheit\humanmedizin\allgemeinmedizin\PRIMA-eDS\work%20packages%20(WP%2001-%20WP%2012)\WP_02%20SR_recommend\01-plat%20agr%20inhibitors_all%20indic\8-%20Publication\submission%203\Anne\PAI_main%20manuscript_ohne%20Tabellen.doc#_ENREF_85)] | |  |  |  |  |  |  |  |  |  |  |  |  |  |  |  |  |  |  |  |  | x |  |
| WATCH 2009 [[86](file:///T:\gesundheit\humanmedizin\allgemeinmedizin\PRIMA-eDS\work%20packages%20(WP%2001-%20WP%2012)\WP_02%20SR_recommend\01-plat%20agr%20inhibitors_all%20indic\8-%20Publication\submission%203\Anne\PAI_main%20manuscript_ohne%20Tabellen.doc#_ENREF_86)] | |  |  |  |  |  |  |  |  |  |  |  |  |  |  |  |  |  |  |  |  | x |  |
| ENGAGE AF-TIMI 2013 [[87](file:///T:\gesundheit\humanmedizin\allgemeinmedizin\PRIMA-eDS\work%20packages%20(WP%2001-%20WP%2012)\WP_02%20SR_recommend\01-plat%20agr%20inhibitors_all%20indic\8-%20Publication\submission%203\Anne\PAI_main%20manuscript_ohne%20Tabellen.doc#_ENREF_87)] | |  |  |  |  |  | x |  |  |  |  |  |  |  |  |  |  | x |  |  |  |  |  |
| ARISTOTLE 2011 [[88](file:///T:\gesundheit\humanmedizin\allgemeinmedizin\PRIMA-eDS\work%20packages%20(WP%2001-%20WP%2012)\WP_02%20SR_recommend\01-plat%20agr%20inhibitors_all%20indic\8-%20Publication\submission%203\Anne\PAI_main%20manuscript_ohne%20Tabellen.doc#_ENREF_88)] | |  |  |  | x |  | x |  |  |  | x |  |  |  |  |  |  | x |  |  |  |  |  |
| ARISTOTLE-J 2011 [[89](file:///T:\gesundheit\humanmedizin\allgemeinmedizin\PRIMA-eDS\work%20packages%20(WP%2001-%20WP%2012)\WP_02%20SR_recommend\01-plat%20agr%20inhibitors_all%20indic\8-%20Publication\submission%203\Anne\PAI_main%20manuscript_ohne%20Tabellen.doc#_ENREF_89)] | |  |  |  | x |  | x |  |  |  |  |  |  |  |  |  |  | x |  |  |  |  |  |
| RE-LY 2009 [[90](file:///T:\gesundheit\humanmedizin\allgemeinmedizin\PRIMA-eDS\work%20packages%20(WP%2001-%20WP%2012)\WP_02%20SR_recommend\01-plat%20agr%20inhibitors_all%20indic\8-%20Publication\submission%203\Anne\PAI_main%20manuscript_ohne%20Tabellen.doc#_ENREF_90)] | |  |  |  | x |  | x |  | x |  | x |  |  |  |  |  |  | x |  |  |  |  |  |
| ROCKET-AF 2011 [[91](file:///T:\gesundheit\humanmedizin\allgemeinmedizin\PRIMA-eDS\work%20packages%20(WP%2001-%20WP%2012)\WP_02%20SR_recommend\01-plat%20agr%20inhibitors_all%20indic\8-%20Publication\submission%203\Anne\PAI_main%20manuscript_ohne%20Tabellen.doc#_ENREF_91)] | |  |  |  | x |  | x |  |  |  | x |  |  |  |  |  |  | x |  |  |  |  |  |
| J-ROCKET 2014 [[92](file:///T:\gesundheit\humanmedizin\allgemeinmedizin\PRIMA-eDS\work%20packages%20(WP%2001-%20WP%2012)\WP_02%20SR_recommend\01-plat%20agr%20inhibitors_all%20indic\8-%20Publication\submission%203\Anne\PAI_main%20manuscript_ohne%20Tabellen.doc#_ENREF_92)] | |  |  |  |  |  |  |  |  |  |  |  |  |  |  |  |  | x |  |  |  |  |  |
| JAST 2006 [[93](file:///T:\gesundheit\humanmedizin\allgemeinmedizin\PRIMA-eDS\work%20packages%20(WP%2001-%20WP%2012)\WP_02%20SR_recommend\01-plat%20agr%20inhibitors_all%20indic\8-%20Publication\submission%203\Anne\PAI_main%20manuscript_ohne%20Tabellen.doc#_ENREF_93)] | |  |  |  | x |  | x |  | x |  | x |  |  | x |  |  |  | x |  |  |  |  |  |
| PETRO 2007 [[94](file:///T:\gesundheit\humanmedizin\allgemeinmedizin\PRIMA-eDS\work%20packages%20(WP%2001-%20WP%2012)\WP_02%20SR_recommend\01-plat%20agr%20inhibitors_all%20indic\8-%20Publication\submission%203\Anne\PAI_main%20manuscript_ohne%20Tabellen.doc#_ENREF_94)] | |  |  |  | x |  | x (not included in analysis) |  |  |  |  |  |  | x |  |  |  | x |  |  |  |  |  |
| AVERROES 2011[[95](file:///T:\gesundheit\humanmedizin\allgemeinmedizin\PRIMA-eDS\work%20packages%20(WP%2001-%20WP%2012)\WP_02%20SR_recommend\01-plat%20agr%20inhibitors_all%20indic\8-%20Publication\submission%203\Anne\PAI_main%20manuscript_ohne%20Tabellen.doc#_ENREF_95)] | |  |  |  | x |  | x |  |  |  | x |  |  |  |  |  |  | x |  |  |  |  |  |
| NCT 2007 | |  |  |  |  |  |  |  |  |  |  |  |  |  |  |  |  | x |  |  |  |  |  |
| ACTIVE A 2009 [[96](file:///T:\gesundheit\humanmedizin\allgemeinmedizin\PRIMA-eDS\work%20packages%20(WP%2001-%20WP%2012)\WP_02%20SR_recommend\01-plat%20agr%20inhibitors_all%20indic\8-%20Publication\submission%203\Anne\PAI_main%20manuscript_ohne%20Tabellen.doc#_ENREF_96)] | |  |  |  | x |  | x |  | x |  | x |  |  |  |  |  |  | x |  |  |  |  | x |
| MWNAF  1998 [[97](file:///T:\gesundheit\humanmedizin\allgemeinmedizin\PRIMA-eDS\work%20packages%20(WP%2001-%20WP%2012)\WP_02%20SR_recommend\01-plat%20agr%20inhibitors_all%20indic\8-%20Publication\submission%203\Anne\PAI_main%20manuscript_ohne%20Tabellen.doc#_ENREF_97)] | |  |  | x |  |  |  | x |  |  |  |  |  | x |  |  |  |  | x |  |  |  |  |
| SPORTIF II 2003 [[98](file:///T:\gesundheit\humanmedizin\allgemeinmedizin\PRIMA-eDS\work%20packages%20(WP%2001-%20WP%2012)\WP_02%20SR_recommend\01-plat%20agr%20inhibitors_all%20indic\8-%20Publication\submission%203\Anne\PAI_main%20manuscript_ohne%20Tabellen.doc#_ENREF_98)] | |  |  |  |  |  |  |  |  |  |  |  |  | x |  |  |  |  |  |  |  |  |  |
| SPORTIF III 2003 [[99](file:///T:\gesundheit\humanmedizin\allgemeinmedizin\PRIMA-eDS\work%20packages%20(WP%2001-%20WP%2012)\WP_02%20SR_recommend\01-plat%20agr%20inhibitors_all%20indic\8-%20Publication\submission%203\Anne\PAI_main%20manuscript_ohne%20Tabellen.doc#_ENREF_99)] | |  |  |  |  |  |  | x | x |  |  |  |  | x |  |  |  |  | x |  |  |  |  |
| SPORTIF V 2005 [[100](file:///T:\gesundheit\humanmedizin\allgemeinmedizin\PRIMA-eDS\work%20packages%20(WP%2001-%20WP%2012)\WP_02%20SR_recommend\01-plat%20agr%20inhibitors_all%20indic\8-%20Publication\submission%203\Anne\PAI_main%20manuscript_ohne%20Tabellen.doc#_ENREF_100)] | |  |  |  |  |  |  | x | x |  |  |  |  | x |  |  |  |  | x |  |  |  |  |
| LASAF 1999 [[101](file:///T:\gesundheit\humanmedizin\allgemeinmedizin\PRIMA-eDS\work%20packages%20(WP%2001-%20WP%2012)\WP_02%20SR_recommend\01-plat%20agr%20inhibitors_all%20indic\8-%20Publication\submission%203\Anne\PAI_main%20manuscript_ohne%20Tabellen.doc#_ENREF_101)] | |  |  |  | x |  |  | x |  |  | x |  |  | x |  |  |  | x |  |  |  |  |  |
| FFAACS 2001 [[102](file:///T:\gesundheit\humanmedizin\allgemeinmedizin\PRIMA-eDS\work%20packages%20(WP%2001-%20WP%2012)\WP_02%20SR_recommend\01-plat%20agr%20inhibitors_all%20indic\8-%20Publication\submission%203\Anne\PAI_main%20manuscript_ohne%20Tabellen.doc#_ENREF_102)] | |  |  |  |  |  |  |  | x |  |  |  |  | x |  |  |  |  |  |  |  |  |  |
| JNAFESP 2000 [[103](file:///T:\gesundheit\humanmedizin\allgemeinmedizin\PRIMA-eDS\work%20packages%20(WP%2001-%20WP%2012)\WP_02%20SR_recommend\01-plat%20agr%20inhibitors_all%20indic\8-%20Publication\submission%203\Anne\PAI_main%20manuscript_ohne%20Tabellen.doc#_ENREF_103)] | |  |  | x |  |  |  | x | x |  |  |  |  | x |  |  |  |  |  |  |  |  |  |
| SAPAT1992 [[104](file:///T:\gesundheit\humanmedizin\allgemeinmedizin\PRIMA-eDS\work%20packages%20(WP%2001-%20WP%2012)\WP_02%20SR_recommend\01-plat%20agr%20inhibitors_all%20indic\8-%20Publication\submission%203\Anne\PAI_main%20manuscript_ohne%20Tabellen.doc#_ENREF_104)] | |  |  |  |  |  |  |  |  | x |  |  |  |  |  | x |  |  |  |  |  |  |  |
| JAFT2006 [[93](file:///T:\gesundheit\humanmedizin\allgemeinmedizin\PRIMA-eDS\work%20packages%20(WP%2001-%20WP%2012)\WP_02%20SR_recommend\01-plat%20agr%20inhibitors_all%20indic\8-%20Publication\submission%203\Anne\PAI_main%20manuscript_ohne%20Tabellen.doc#_ENREF_93)] | |  |  |  |  |  |  |  |  | x |  |  |  |  |  |  |  |  |  |  |  |  |  |
| JPAD 2008 [33] | |  |  |  |  |  |  |  |  | x |  |  |  |  |  |  |  |  |  |  |  |  |  |
| ESPS 1990 [[105](file:///T:\gesundheit\humanmedizin\allgemeinmedizin\PRIMA-eDS\work%20packages%20(WP%2001-%20WP%2012)\WP_02%20SR_recommend\01-plat%20agr%20inhibitors_all%20indic\8-%20Publication\submission%203\Anne\PAI_main%20manuscript_ohne%20Tabellen.doc#_ENREF_105)] | |  |  |  |  |  |  |  |  |  |  |  |  |  |  |  | x |  |  |  |  |  |  |
| ESPS II 1996 [[106](file:///T:\gesundheit\humanmedizin\allgemeinmedizin\PRIMA-eDS\work%20packages%20(WP%2001-%20WP%2012)\WP_02%20SR_recommend\01-plat%20agr%20inhibitors_all%20indic\8-%20Publication\submission%203\Anne\PAI_main%20manuscript_ohne%20Tabellen.doc#_ENREF_106)] | |  |  |  |  |  |  |  |  | x |  |  | x | x |  |  | x |  |  |  |  |  |  |
| HOT 1998 [[107](file:///T:\gesundheit\humanmedizin\allgemeinmedizin\PRIMA-eDS\work%20packages%20(WP%2001-%20WP%2012)\WP_02%20SR_recommend\01-plat%20agr%20inhibitors_all%20indic\8-%20Publication\submission%203\Anne\PAI_main%20manuscript_ohne%20Tabellen.doc#_ENREF_107)] | |  |  |  |  | x |  |  |  | x |  |  |  |  |  |  |  |  |  |  |  |  |  |
| AAAT 2010 [[108](file:///T:\gesundheit\humanmedizin\allgemeinmedizin\PRIMA-eDS\work%20packages%20(WP%2001-%20WP%2012)\WP_02%20SR_recommend\01-plat%20agr%20inhibitors_all%20indic\8-%20Publication\submission%203\Anne\PAI_main%20manuscript_ohne%20Tabellen.doc#_ENREF_108)] | |  |  |  |  |  |  |  |  | x |  |  |  |  |  |  |  |  |  |  |  |  |  |
| AICLA 1983 [[109](file:///T:\gesundheit\humanmedizin\allgemeinmedizin\PRIMA-eDS\work%20packages%20(WP%2001-%20WP%2012)\WP_02%20SR_recommend\01-plat%20agr%20inhibitors_all%20indic\8-%20Publication\submission%203\Anne\PAI_main%20manuscript_ohne%20Tabellen.doc#_ENREF_109)] | |  |  |  |  |  |  |  |  |  |  |  | x |  |  | x | x |  |  |  |  |  |  |
| ACSSG 1985 [[110](file:///T:\gesundheit\humanmedizin\allgemeinmedizin\PRIMA-eDS\work%20packages%20(WP%2001-%20WP%2012)\WP_02%20SR_recommend\01-plat%20agr%20inhibitors_all%20indic\8-%20Publication\submission%203\Anne\PAI_main%20manuscript_ohne%20Tabellen.doc#_ENREF_110)] | |  |  |  |  |  |  |  |  |  |  |  | x |  |  |  | x |  |  |  |  |  |  |
| CASCADE 2010 [[111](file:///T:\gesundheit\humanmedizin\allgemeinmedizin\PRIMA-eDS\work%20packages%20(WP%2001-%20WP%2012)\WP_02%20SR_recommend\01-plat%20agr%20inhibitors_all%20indic\8-%20Publication\submission%203\Anne\PAI_main%20manuscript_ohne%20Tabellen.doc#_ENREF_111)] | |  |  |  |  |  |  |  |  |  |  |  |  |  |  |  |  |  |  |  |  |  | x |
| CHARISMA 2006 [[112](file:///T:\gesundheit\humanmedizin\allgemeinmedizin\PRIMA-eDS\work%20packages%20(WP%2001-%20WP%2012)\WP_02%20SR_recommend\01-plat%20agr%20inhibitors_all%20indic\8-%20Publication\submission%203\Anne\PAI_main%20manuscript_ohne%20Tabellen.doc#_ENREF_112)] | |  |  |  |  |  |  |  |  |  |  |  |  |  |  |  |  |  |  |  |  |  | x |
| CREDO 2002 [[113](file:///T:\gesundheit\humanmedizin\allgemeinmedizin\PRIMA-eDS\work%20packages%20(WP%2001-%20WP%2012)\WP_02%20SR_recommend\01-plat%20agr%20inhibitors_all%20indic\8-%20Publication\submission%203\Anne\PAI_main%20manuscript_ohne%20Tabellen.doc#_ENREF_113)] | |  |  |  |  |  |  |  |  |  |  |  |  |  |  |  |  |  |  |  |  |  | x |
| MATCH 2004 [53] | |  |  |  |  |  |  |  |  |  |  |  |  |  |  |  |  |  |  |  |  |  | x |
| ESPRIT 2006 [[114](file:///T:\gesundheit\humanmedizin\allgemeinmedizin\PRIMA-eDS\work%20packages%20(WP%2001-%20WP%2012)\WP_02%20SR_recommend\01-plat%20agr%20inhibitors_all%20indic\8-%20Publication\submission%203\Anne\PAI_main%20manuscript_ohne%20Tabellen.doc#_ENREF_114)] | |  |  |  |  |  |  |  |  |  |  |  | x |  |  |  |  |  |  |  |  |  |  |
| Chinese/ATAFS 2006 [[115](file:///T:\gesundheit\humanmedizin\allgemeinmedizin\PRIMA-eDS\work%20packages%20(WP%2001-%20WP%2012)\WP_02%20SR_recommend\01-plat%20agr%20inhibitors_all%20indic\8-%20Publication\submission%203\Anne\PAI_main%20manuscript_ohne%20Tabellen.doc#_ENREF_115)] | |  |  | x |  |  |  |  |  |  | x |  |  | x |  |  |  |  |  |  |  |  |  |
| UK-TIA 1991 [[116](file:///T:\gesundheit\humanmedizin\allgemeinmedizin\PRIMA-eDS\work%20packages%20(WP%2001-%20WP%2012)\WP_02%20SR_recommend\01-plat%20agr%20inhibitors_all%20indic\8-%20Publication\submission%203\Anne\PAI_main%20manuscript_ohne%20Tabellen.doc#_ENREF_116)] | |  |  |  |  |  |  |  |  |  |  |  |  | x |  | x |  |  |  |  |  |  |  |
| SAFT 2003 [[117](file:///T:\gesundheit\humanmedizin\allgemeinmedizin\PRIMA-eDS\work%20packages%20(WP%2001-%20WP%2012)\WP_02%20SR_recommend\01-plat%20agr%20inhibitors_all%20indic\8-%20Publication\submission%203\Anne\PAI_main%20manuscript_ohne%20Tabellen.doc#_ENREF_117)] | |  |  |  | x |  |  | x (included in initial analysis but excluded in sensivity analysis) |  |  |  |  |  | x |  |  |  |  |  |  |  |  |  |
| SALT Collaborative Group 1991 [[118](file:///T:\gesundheit\humanmedizin\allgemeinmedizin\PRIMA-eDS\work%20packages%20(WP%2001-%20WP%2012)\WP_02%20SR_recommend\01-plat%20agr%20inhibitors_all%20indic\8-%20Publication\submission%203\Anne\PAI_main%20manuscript_ohne%20Tabellen.doc#_ENREF_118)] | |  |  |  |  |  |  |  |  |  |  |  |  |  |  | x |  |  |  |  |  |  |  |
| MRC 1998 [[119](file:///T:\gesundheit\humanmedizin\allgemeinmedizin\PRIMA-eDS\work%20packages%20(WP%2001-%20WP%2012)\WP_02%20SR_recommend\01-plat%20agr%20inhibitors_all%20indic\8-%20Publication\submission%203\Anne\PAI_main%20manuscript_ohne%20Tabellen.doc#_ENREF_119)] | |  |  |  |  |  |  |  |  |  |  |  |  |  | x |  |  |  |  |  |  |  |  |
| The CURE 2001 [[120](file:///T:\gesundheit\humanmedizin\allgemeinmedizin\PRIMA-eDS\work%20packages%20(WP%2001-%20WP%2012)\WP_02%20SR_recommend\01-plat%20agr%20inhibitors_all%20indic\8-%20Publication\submission%203\Anne\PAI_main%20manuscript_ohne%20Tabellen.doc#_ENREF_120)] | |  |  |  |  |  |  |  |  |  |  |  |  |  |  |  |  |  |  |  |  |  | x |
| SJ Park 2010 [[121](file:///T:\gesundheit\humanmedizin\allgemeinmedizin\PRIMA-eDS\work%20packages%20(WP%2001-%20WP%2012)\WP_02%20SR_recommend\01-plat%20agr%20inhibitors_all%20indic\8-%20Publication\submission%203\Anne\PAI_main%20manuscript_ohne%20Tabellen.doc#_ENREF_121)] | |  |  |  |  |  |  |  |  |  |  |  |  |  |  |  |  |  |  |  |  |  | x |
| Huyn et al. 2001 [36] | |  |  |  |  |  |  |  |  |  |  |  |  |  |  |  |  |  |  |  |  | x |  |
| ISIS-2 Collaborative Group 1988 [[122](file:///T:\gesundheit\humanmedizin\allgemeinmedizin\PRIMA-eDS\work%20packages%20(WP%2001-%20WP%2012)\WP_02%20SR_recommend\01-plat%20agr%20inhibitors_all%20indic\8-%20Publication\submission%203\Anne\PAI_main%20manuscript_ohne%20Tabellen.doc#_ENREF_122)] | |  |  |  |  |  |  |  |  |  |  |  |  |  |  | x |  |  |  |  |  |  |  |
| Colli et al. (WoA Epic) 2007 [[123](file:///T:\gesundheit\humanmedizin\allgemeinmedizin\PRIMA-eDS\work%20packages%20(WP%2001-%20WP%2012)\WP_02%20SR_recommend\01-plat%20agr%20inhibitors_all%20indic\8-%20Publication\submission%203\Anne\PAI_main%20manuscript_ohne%20Tabellen.doc#_ENREF_123)] | |  |  |  |  |  |  |  |  |  |  |  |  |  |  |  |  |  |  |  |  | x |  |
| Evans et al. 2001 [[124](file:///T:\gesundheit\humanmedizin\allgemeinmedizin\PRIMA-eDS\work%20packages%20(WP%2001-%20WP%2012)\WP_02%20SR_recommend\01-plat%20agr%20inhibitors_all%20indic\8-%20Publication\submission%203\Anne\PAI_main%20manuscript_ohne%20Tabellen.doc#_ENREF_124)] | |  |  |  |  |  |  |  |  |  |  |  |  |  |  |  |  |  | (x) |  |  |  |  |
| Caneschi 1985 [[125](file:///T:\gesundheit\humanmedizin\allgemeinmedizin\PRIMA-eDS\work%20packages%20(WP%2001-%20WP%2012)\WP_02%20SR_recommend\01-plat%20agr%20inhibitors_all%20indic\8-%20Publication\submission%203\Anne\PAI_main%20manuscript_ohne%20Tabellen.doc#_ENREF_125)] | |  |  |  |  |  |  |  |  |  |  |  |  |  |  |  | x |  |  |  |  |  |  |
| Acheson 1969 [[126](file:///T:\gesundheit\humanmedizin\allgemeinmedizin\PRIMA-eDS\work%20packages%20(WP%2001-%20WP%2012)\WP_02%20SR_recommend\01-plat%20agr%20inhibitors_all%20indic\8-%20Publication\submission%203\Anne\PAI_main%20manuscript_ohne%20Tabellen.doc#_ENREF_126)] | |  |  |  |  |  |  |  |  |  |  |  |  |  |  |  | x |  |  |  |  |  |  |
| Matias Guiu 1987 [[127](file:///T:\gesundheit\humanmedizin\allgemeinmedizin\PRIMA-eDS\work%20packages%20(WP%2001-%20WP%2012)\WP_02%20SR_recommend\01-plat%20agr%20inhibitors_all%20indic\8-%20Publication\submission%203\Anne\PAI_main%20manuscript_ohne%20Tabellen.doc#_ENREF_127)] | |  |  |  |  |  |  |  |  |  |  |  |  |  |  |  | x (excluded from main analyses) |  |  |  |  |  |  |
| Guiraud-Chaumeil 1982 [[128](file:///T:\gesundheit\humanmedizin\allgemeinmedizin\PRIMA-eDS\work%20packages%20(WP%2001-%20WP%2012)\WP_02%20SR_recommend\01-plat%20agr%20inhibitors_all%20indic\8-%20Publication\submission%203\Anne\PAI_main%20manuscript_ohne%20Tabellen.doc#_ENREF_128)] | |  |  |  |  |  |  |  |  |  |  |  | x |  |  |  | x |  |  |  |  |  |  |
| Fields et al. 1977 [[129](file:///T:\gesundheit\humanmedizin\allgemeinmedizin\PRIMA-eDS\work%20packages%20(WP%2001-%20WP%2012)\WP_02%20SR_recommend\01-plat%20agr%20inhibitors_all%20indic\8-%20Publication\submission%203\Anne\PAI_main%20manuscript_ohne%20Tabellen.doc#_ENREF_129)] | |  |  |  |  |  |  |  |  |  |  |  |  |  |  | x |  |  |  |  |  |  |  |
| Fields et al. 1978 [[130](file:///T:\gesundheit\humanmedizin\allgemeinmedizin\PRIMA-eDS\work%20packages%20(WP%2001-%20WP%2012)\WP_02%20SR_recommend\01-plat%20agr%20inhibitors_all%20indic\8-%20Publication\submission%203\Anne\PAI_main%20manuscript_ohne%20Tabellen.doc#_ENREF_130)] | |  |  |  |  |  |  |  |  |  |  |  |  |  |  | x |  |  |  |  |  |  |  |
| Elwood and Sweetham et al. 1979 [[131](file:///T:\gesundheit\humanmedizin\allgemeinmedizin\PRIMA-eDS\work%20packages%20(WP%2001-%20WP%2012)\WP_02%20SR_recommend\01-plat%20agr%20inhibitors_all%20indic\8-%20Publication\submission%203\Anne\PAI_main%20manuscript_ohne%20Tabellen.doc#_ENREF_131)] | |  |  |  |  |  |  |  |  |  |  |  |  |  |  | x |  |  |  |  |  |  |  |
| Sorensen et al. 1983 [[132](file:///T:\gesundheit\humanmedizin\allgemeinmedizin\PRIMA-eDS\work%20packages%20(WP%2001-%20WP%2012)\WP_02%20SR_recommend\01-plat%20agr%20inhibitors_all%20indic\8-%20Publication\submission%203\Anne\PAI_main%20manuscript_ohne%20Tabellen.doc#_ENREF_132)] | |  |  |  |  |  |  |  |  |  |  |  |  |  |  | x |  |  |  |  |  |  |  |
| Britton et al.1987 [58] | |  |  |  |  |  |  |  |  |  |  |  |  |  |  | x |  |  |  |  |  |  |  |
| Cote et al. 1995 [[133](file:///T:\gesundheit\humanmedizin\allgemeinmedizin\PRIMA-eDS\work%20packages%20(WP%2001-%20WP%2012)\WP_02%20SR_recommend\01-plat%20agr%20inhibitors_all%20indic\8-%20Publication\submission%203\Anne\PAI_main%20manuscript_ohne%20Tabellen.doc#_ENREF_133)] | |  |  |  |  |  |  |  |  |  |  |  |  |  |  | x |  |  |  |  |  |  |  |
| Turpie et al. 1993 [[134](file:///T:\gesundheit\humanmedizin\allgemeinmedizin\PRIMA-eDS\work%20packages%20(WP%2001-%20WP%2012)\WP_02%20SR_recommend\01-plat%20agr%20inhibitors_all%20indic\8-%20Publication\submission%203\Anne\PAI_main%20manuscript_ohne%20Tabellen.doc#_ENREF_134)] | |  |  |  |  |  |  |  |  |  |  |  |  |  | x |  |  |  |  |  |  |  |  |
| Chesebro et al. 1983 [[135](file:///T:\gesundheit\humanmedizin\allgemeinmedizin\PRIMA-eDS\work%20packages%20(WP%2001-%20WP%2012)\WP_02%20SR_recommend\01-plat%20agr%20inhibitors_all%20indic\8-%20Publication\submission%203\Anne\PAI_main%20manuscript_ohne%20Tabellen.doc#_ENREF_135)] | |  |  |  |  |  |  |  |  |  |  |  |  |  | x |  |  |  |  |  |  |  |  |
| Altman et al. 1976 [[136](file:///T:\gesundheit\humanmedizin\allgemeinmedizin\PRIMA-eDS\work%20packages%20(WP%2001-%20WP%2012)\WP_02%20SR_recommend\01-plat%20agr%20inhibitors_all%20indic\8-%20Publication\submission%203\Anne\PAI_main%20manuscript_ohne%20Tabellen.doc#_ENREF_136)] | |  |  |  |  |  |  |  |  |  |  |  |  |  | x |  |  |  |  |  |  |  |  |
| Dale et al. 1980 [[137](file:///T:\gesundheit\humanmedizin\allgemeinmedizin\PRIMA-eDS\work%20packages%20(WP%2001-%20WP%2012)\WP_02%20SR_recommend\01-plat%20agr%20inhibitors_all%20indic\8-%20Publication\submission%203\Anne\PAI_main%20manuscript_ohne%20Tabellen.doc#_ENREF_137)] | |  |  |  |  |  |  |  |  |  |  |  |  |  | x |  |  |  |  |  |  |  |  |
| Harenberg et al. 1993 [[138](file:///T:\gesundheit\humanmedizin\allgemeinmedizin\PRIMA-eDS\work%20packages%20(WP%2001-%20WP%2012)\WP_02%20SR_recommend\01-plat%20agr%20inhibitors_all%20indic\8-%20Publication\submission%203\Anne\PAI_main%20manuscript_ohne%20Tabellen.doc#_ENREF_138)] | |  |  |  |  |  |  |  |  |  |  |  |  | x |  |  |  |  |  | x |  |  |  |
| British Doctor study Peto et al. 1988 [[139](file:///T:\gesundheit\humanmedizin\allgemeinmedizin\PRIMA-eDS\work%20packages%20(WP%2001-%20WP%2012)\WP_02%20SR_recommend\01-plat%20agr%20inhibitors_all%20indic\8-%20Publication\submission%203\Anne\PAI_main%20manuscript_ohne%20Tabellen.doc#_ENREF_139)] | |  |  |  |  | x |  |  |  |  |  |  |  |  |  | x |  |  |  |  |  |  |  |
| Liu et al. 2014 [47] | |  |  |  |  |  |  |  |  |  |  |  |  |  |  |  |  | x |  |  |  |  |  |
| Weitz et al. 2012 [[140](file:///T:\gesundheit\humanmedizin\allgemeinmedizin\PRIMA-eDS\work%20packages%20(WP%2001-%20WP%2012)\WP_02%20SR_recommend\01-plat%20agr%20inhibitors_all%20indic\8-%20Publication\submission%203\Anne\PAI_main%20manuscript_ohne%20Tabellen.doc#_ENREF_140)] | |  |  |  | x |  |  |  |  |  |  |  |  |  |  |  |  | x |  |  |  |  |  |
| Chung et al. 2011 [[141](file:///T:\gesundheit\humanmedizin\allgemeinmedizin\PRIMA-eDS\work%20packages%20(WP%2001-%20WP%2012)\WP_02%20SR_recommend\01-plat%20agr%20inhibitors_all%20indic\8-%20Publication\submission%203\Anne\PAI_main%20manuscript_ohne%20Tabellen.doc#_ENREF_141)] | |  |  |  | x |  | x (not included in analysis) |  |  |  |  |  |  |  |  |  |  | x |  |  |  |  |  |
| Chang et al. 2015 (unrandomized) [[142](file:///T:\gesundheit\humanmedizin\allgemeinmedizin\PRIMA-eDS\work%20packages%20(WP%2001-%20WP%2012)\WP_02%20SR_recommend\01-plat%20agr%20inhibitors_all%20indic\8-%20Publication\submission%203\Anne\PAI_main%20manuscript_ohne%20Tabellen.doc#_ENREF_142)] | |  |  |  |  |  |  |  |  |  |  |  |  |  |  |  |  | x |  |  |  |  |  |
| Ellis et al. 2014/ Lleva 2009 (unrandomized) [[143](file:///T:\gesundheit\humanmedizin\allgemeinmedizin\PRIMA-eDS\work%20packages%20(WP%2001-%20WP%2012)\WP_02%20SR_recommend\01-plat%20agr%20inhibitors_all%20indic\8-%20Publication\submission%203\Anne\PAI_main%20manuscript_ohne%20Tabellen.doc#_ENREF_143)] | |  |  |  |  |  |  |  |  |  |  |  |  |  |  |  |  | x |  |  |  |  |  |
| Abraham et al. 2015 (unrandomized) [[144](file:///T:\gesundheit\humanmedizin\allgemeinmedizin\PRIMA-eDS\work%20packages%20(WP%2001-%20WP%2012)\WP_02%20SR_recommend\01-plat%20agr%20inhibitors_all%20indic\8-%20Publication\submission%203\Anne\PAI_main%20manuscript_ohne%20Tabellen.doc#_ENREF_144)] | |  |  |  |  |  |  |  |  |  |  |  |  |  |  |  |  | x |  |  |  |  |  |
| Graham et al. 2014 (unrandomized) [[145](file:///T:\gesundheit\humanmedizin\allgemeinmedizin\PRIMA-eDS\work%20packages%20(WP%2001-%20WP%2012)\WP_02%20SR_recommend\01-plat%20agr%20inhibitors_all%20indic\8-%20Publication\submission%203\Anne\PAI_main%20manuscript_ohne%20Tabellen.doc#_ENREF_145)] | |  |  |  |  |  |  |  |  |  |  |  |  |  |  |  |  | x |  |  |  |  |  |
| Fontaine et al. 2014 (unrandomized) [[146](file:///T:\gesundheit\humanmedizin\allgemeinmedizin\PRIMA-eDS\work%20packages%20(WP%2001-%20WP%2012)\WP_02%20SR_recommend\01-plat%20agr%20inhibitors_all%20indic\8-%20Publication\submission%203\Anne\PAI_main%20manuscript_ohne%20Tabellen.doc#_ENREF_146)] | |  |  |  |  |  |  |  |  |  |  |  |  |  |  |  |  | x |  |  |  |  |  |
| Strunets et al. 2014 (unrandomized) [[147](file:///T:\gesundheit\humanmedizin\allgemeinmedizin\PRIMA-eDS\work%20packages%20(WP%2001-%20WP%2012)\WP_02%20SR_recommend\01-plat%20agr%20inhibitors_all%20indic\8-%20Publication\submission%203\Anne\PAI_main%20manuscript_ohne%20Tabellen.doc#_ENREF_147)] | |  |  |  |  |  |  |  |  |  |  |  |  |  |  |  |  | x |  |  |  |  |  |
| Bengtson et al. 2014 (unrandomized) [[148](file:///T:\gesundheit\humanmedizin\allgemeinmedizin\PRIMA-eDS\work%20packages%20(WP%2001-%20WP%2012)\WP_02%20SR_recommend\01-plat%20agr%20inhibitors_all%20indic\8-%20Publication\submission%203\Anne\PAI_main%20manuscript_ohne%20Tabellen.doc#_ENREF_148)] | |  |  |  |  |  |  |  |  |  |  |  |  |  |  |  |  | x |  |  |  |  |  |
| Hernandez et al. 2014 (unrandomized) [[149](file:///T:\gesundheit\humanmedizin\allgemeinmedizin\PRIMA-eDS\work%20packages%20(WP%2001-%20WP%2012)\WP_02%20SR_recommend\01-plat%20agr%20inhibitors_all%20indic\8-%20Publication\submission%203\Anne\PAI_main%20manuscript_ohne%20Tabellen.doc#_ENREF_149)] | |  |  |  |  |  |  |  |  |  |  |  |  |  |  |  |  | x |  |  |  |  |  |
| Quintanilla et al. 2014 (unrandomized) [[150](file:///T:\gesundheit\humanmedizin\allgemeinmedizin\PRIMA-eDS\work%20packages%20(WP%2001-%20WP%2012)\WP_02%20SR_recommend\01-plat%20agr%20inhibitors_all%20indic\8-%20Publication\submission%203\Anne\PAI_main%20manuscript_ohne%20Tabellen.doc#_ENREF_150)] | |  |  |  |  |  |  |  |  |  |  |  |  |  |  |  |  | x |  |  |  |  |  |
| Lauffenburger et al. 2015 (unrandomized) [[151](file:///T:\gesundheit\humanmedizin\allgemeinmedizin\PRIMA-eDS\work%20packages%20(WP%2001-%20WP%2012)\WP_02%20SR_recommend\01-plat%20agr%20inhibitors_all%20indic\8-%20Publication\submission%203\Anne\PAI_main%20manuscript_ohne%20Tabellen.doc#_ENREF_151)] | |  |  |  |  |  |  |  |  |  |  |  |  |  |  |  |  | x |  |  |  |  |  |
| Staerk et al. 2015 (unrandomized) [[152](file:///T:\gesundheit\humanmedizin\allgemeinmedizin\PRIMA-eDS\work%20packages%20(WP%2001-%20WP%2012)\WP_02%20SR_recommend\01-plat%20agr%20inhibitors_all%20indic\8-%20Publication\submission%203\Anne\PAI_main%20manuscript_ohne%20Tabellen.doc#_ENREF_152)] | |  |  |  |  |  |  |  |  |  |  |  |  |  |  |  |  | x |  |  |  |  |  |
| Larsen et al. 2014 (unrandomized) [[153](file:///T:\gesundheit\humanmedizin\allgemeinmedizin\PRIMA-eDS\work%20packages%20(WP%2001-%20WP%2012)\WP_02%20SR_recommend\01-plat%20agr%20inhibitors_all%20indic\8-%20Publication\submission%203\Anne\PAI_main%20manuscript_ohne%20Tabellen.doc#_ENREF_153)] | |  |  |  |  |  |  |  |  |  |  |  |  |  |  |  |  | x |  |  |  |  |  |
| Larsen et al. 2014 (unrandomized) [[154](file:///T:\gesundheit\humanmedizin\allgemeinmedizin\PRIMA-eDS\work%20packages%20(WP%2001-%20WP%2012)\WP_02%20SR_recommend\01-plat%20agr%20inhibitors_all%20indic\8-%20Publication\submission%203\Anne\PAI_main%20manuscript_ohne%20Tabellen.doc#_ENREF_154)] | |  |  |  |  |  |  |  |  |  |  |  |  |  |  |  |  | x |  |  |  |  |  |
| Larsen et al. 2013 (unrandomized) [[155](file:///T:\gesundheit\humanmedizin\allgemeinmedizin\PRIMA-eDS\work%20packages%20(WP%2001-%20WP%2012)\WP_02%20SR_recommend\01-plat%20agr%20inhibitors_all%20indic\8-%20Publication\submission%203\Anne\PAI_main%20manuscript_ohne%20Tabellen.doc#_ENREF_155)] | |  |  |  |  |  |  |  |  |  |  |  |  |  |  |  |  | x |  |  |  |  |  |
| Sorensen et al. 2013 (unrandomized) [[156](file:///T:\gesundheit\humanmedizin\allgemeinmedizin\PRIMA-eDS\work%20packages%20(WP%2001-%20WP%2012)\WP_02%20SR_recommend\01-plat%20agr%20inhibitors_all%20indic\8-%20Publication\submission%203\Anne\PAI_main%20manuscript_ohne%20Tabellen.doc#_ENREF_156)] | |  |  |  |  |  |  |  |  |  |  |  |  |  |  |  |  | x |  |  |  |  |  |
| Tsadok et al. 2013 / Kantharina (unrandomized) [[157](file:///T:\gesundheit\humanmedizin\allgemeinmedizin\PRIMA-eDS\work%20packages%20(WP%2001-%20WP%2012)\WP_02%20SR_recommend\01-plat%20agr%20inhibitors_all%20indic\8-%20Publication\submission%203\Anne\PAI_main%20manuscript_ohne%20Tabellen.doc#_ENREF_157)] | |  |  |  |  |  |  |  |  |  |  |  |  |  |  |  |  | x |  |  |  |  |  |
| Charland et al. 2012 / Holmes (unrandomized) [[158](file:///T:\gesundheit\humanmedizin\allgemeinmedizin\PRIMA-eDS\work%20packages%20(WP%2001-%20WP%2012)\WP_02%20SR_recommend\01-plat%20agr%20inhibitors_all%20indic\8-%20Publication\submission%203\Anne\PAI_main%20manuscript_ohne%20Tabellen.doc#_ENREF_158)] | |  |  |  |  |  |  |  |  |  |  |  |  |  |  |  |  | x |  |  |  |  |  |
| Thelus et al. 2012 / Shalimar (unrandomized) [[159](file:///T:\gesundheit\humanmedizin\allgemeinmedizin\PRIMA-eDS\work%20packages%20(WP%2001-%20WP%2012)\WP_02%20SR_recommend\01-plat%20agr%20inhibitors_all%20indic\8-%20Publication\submission%203\Anne\PAI_main%20manuscript_ohne%20Tabellen.doc#_ENREF_159)] | |  |  |  |  |  |  |  |  |  |  |  |  |  |  |  |  | x |  |  |  |  |  |
| Ho et al. 2012 (unrandomized) [[160](file:///T:\gesundheit\humanmedizin\allgemeinmedizin\PRIMA-eDS\work%20packages%20(WP%2001-%20WP%2012)\WP_02%20SR_recommend\01-plat%20agr%20inhibitors_all%20indic\8-%20Publication\submission%203\Anne\PAI_main%20manuscript_ohne%20Tabellen.doc#_ENREF_160)] | |  |  |  |  |  |  |  |  |  |  |  |  |  |  |  |  | x |  |  |  |  |  |
| Laliberte et al. 2014 (unrandomized) [[161](file:///T:\gesundheit\humanmedizin\allgemeinmedizin\PRIMA-eDS\work%20packages%20(WP%2001-%20WP%2012)\WP_02%20SR_recommend\01-plat%20agr%20inhibitors_all%20indic\8-%20Publication\submission%203\Anne\PAI_main%20manuscript_ohne%20Tabellen.doc#_ENREF_161)] | |  |  |  |  |  |  |  |  |  |  |  |  |  |  |  |  | x |  |  |  |  |  |
| Forslund et al. 2014 (unrandomized) [[162](file:///T:\gesundheit\humanmedizin\allgemeinmedizin\PRIMA-eDS\work%20packages%20(WP%2001-%20WP%2012)\WP_02%20SR_recommend\01-plat%20agr%20inhibitors_all%20indic\8-%20Publication\submission%203\Anne\PAI_main%20manuscript_ohne%20Tabellen.doc#_ENREF_162)] | |  |  |  |  |  |  |  |  |  |  |  |  |  |  |  |  | x |  |  |  |  |  |
| Hansen et al. 2010 (unrandomized) [[163](file:///T:\gesundheit\humanmedizin\allgemeinmedizin\PRIMA-eDS\work%20packages%20(WP%2001-%20WP%2012)\WP_02%20SR_recommend\01-plat%20agr%20inhibitors_all%20indic\8-%20Publication\submission%203\Anne\PAI_main%20manuscript_ohne%20Tabellen.doc#_ENREF_163)] | |  |  |  |  |  |  |  |  |  |  |  |  |  |  |  |  | x |  |  |  |  |  |
| Lip et al. 2015 (unrandomized) [[164](file:///T:\gesundheit\humanmedizin\allgemeinmedizin\PRIMA-eDS\work%20packages%20(WP%2001-%20WP%2012)\WP_02%20SR_recommend\01-plat%20agr%20inhibitors_all%20indic\8-%20Publication\submission%203\Anne\PAI_main%20manuscript_ohne%20Tabellen.doc#_ENREF_164)] | |  |  |  |  |  |  |  |  |  |  |  |  |  |  |  |  | x |  |  |  |  |  |
| An et al./ Richard 2014 (unrandomized) [[165](file:///T:\gesundheit\humanmedizin\allgemeinmedizin\PRIMA-eDS\work%20packages%20(WP%2001-%20WP%2012)\WP_02%20SR_recommend\01-plat%20agr%20inhibitors_all%20indic\8-%20Publication\submission%203\Anne\PAI_main%20manuscript_ohne%20Tabellen.doc#_ENREF_165)] | |  |  |  |  |  |  |  |  |  |  |  |  |  |  |  |  | x |  |  |  |  |  |
| Yamashita et al. 2012 [[166](file:///T:\gesundheit\humanmedizin\allgemeinmedizin\PRIMA-eDS\work%20packages%20(WP%2001-%20WP%2012)\WP_02%20SR_recommend\01-plat%20agr%20inhibitors_all%20indic\8-%20Publication\submission%203\Anne\PAI_main%20manuscript_ohne%20Tabellen.doc#_ENREF_166)] | |  |  |  | x |  | x (not included in analysis) |  |  |  |  |  |  |  |  |  |  | x |  |  |  |  |  |
| Stabile et al. (SAT-TAVI) 2014 [[167](file:///T:\gesundheit\humanmedizin\allgemeinmedizin\PRIMA-eDS\work%20packages%20(WP%2001-%20WP%2012)\WP_02%20SR_recommend\01-plat%20agr%20inhibitors_all%20indic\8-%20Publication\submission%203\Anne\PAI_main%20manuscript_ohne%20Tabellen.doc#_ENREF_167)] | |  |  |  |  |  |  |  |  |  |  | x |  |  |  |  |  |  |  |  |  |  |  |
| Durand et al. 2014 [[168](file:///T:\gesundheit\humanmedizin\allgemeinmedizin\PRIMA-eDS\work%20packages%20(WP%2001-%20WP%2012)\WP_02%20SR_recommend\01-plat%20agr%20inhibitors_all%20indic\8-%20Publication\submission%203\Anne\PAI_main%20manuscript_ohne%20Tabellen.doc#_ENREF_168)] | |  |  |  |  |  |  |  |  |  |  | x |  |  |  |  |  |  |  |  |  |  |  |
| Poliacikova 2013 [[169](file:///T:\gesundheit\humanmedizin\allgemeinmedizin\PRIMA-eDS\work%20packages%20(WP%2001-%20WP%2012)\WP_02%20SR_recommend\01-plat%20agr%20inhibitors_all%20indic\8-%20Publication\submission%203\Anne\PAI_main%20manuscript_ohne%20Tabellen.doc#_ENREF_169)] | |  |  |  |  |  |  |  |  |  |  | x |  |  |  |  |  |  |  |  |  |  |  |
| Ussia 2011 [[170](file:///T:\gesundheit\humanmedizin\allgemeinmedizin\PRIMA-eDS\work%20packages%20(WP%2001-%20WP%2012)\WP_02%20SR_recommend\01-plat%20agr%20inhibitors_all%20indic\8-%20Publication\submission%203\Anne\PAI_main%20manuscript_ohne%20Tabellen.doc#_ENREF_170)] | |  |  |  |  |  |  |  |  |  |  | x |  |  |  |  |  |  |  |  |  |  |  |
| Steering Committee of the Physicians’ Health Study Research Group,1989 [[171](file:///T:\gesundheit\humanmedizin\allgemeinmedizin\PRIMA-eDS\work%20packages%20(WP%2001-%20WP%2012)\WP_02%20SR_recommend\01-plat%20agr%20inhibitors_all%20indic\8-%20Publication\submission%203\Anne\PAI_main%20manuscript_ohne%20Tabellen.doc#_ENREF_171)] | |  |  |  |  |  |  |  |  |  |  |  |  |  |  | x |  |  |  |  |  |  |  |
| US Physicians’ Health Study1989 [[171](file:///T:\gesundheit\humanmedizin\allgemeinmedizin\PRIMA-eDS\work%20packages%20(WP%2001-%20WP%2012)\WP_02%20SR_recommend\01-plat%20agr%20inhibitors_all%20indic\8-%20Publication\submission%203\Anne\PAI_main%20manuscript_ohne%20Tabellen.doc#_ENREF_171)] | |  |  |  |  | x |  |  |  | x |  |  |  |  |  |  |  |  |  |  |  |  |  |
| Thrombosis Prevention Trial 1998 [[119](file:///T:\gesundheit\humanmedizin\allgemeinmedizin\PRIMA-eDS\work%20packages%20(WP%2001-%20WP%2012)\WP_02%20SR_recommend\01-plat%20agr%20inhibitors_all%20indic\8-%20Publication\submission%203\Anne\PAI_main%20manuscript_ohne%20Tabellen.doc#_ENREF_119)] | |  |  |  |  | x |  |  |  |  |  |  |  |  |  |  |  |  |  |  |  |  |  |
| Primary Prevention Project 2001 [[172](file:///T:\gesundheit\humanmedizin\allgemeinmedizin\PRIMA-eDS\work%20packages%20(WP%2001-%20WP%2012)\WP_02%20SR_recommend\01-plat%20agr%20inhibitors_all%20indic\8-%20Publication\submission%203\Anne\PAI_main%20manuscript_ohne%20Tabellen.doc#_ENREF_172)] |  | |  |  |  | x |  |  |  |  |  |  |  |  |  |  |  |  |  |  |  |  |  |
| Women’s Health Study 2005 [[173](file:///T:\gesundheit\humanmedizin\allgemeinmedizin\PRIMA-eDS\work%20packages%20(WP%2001-%20WP%2012)\WP_02%20SR_recommend\01-plat%20agr%20inhibitors_all%20indic\8-%20Publication\submission%203\Anne\PAI_main%20manuscript_ohne%20Tabellen.doc#_ENREF_173)] |  | |  |  |  | x |  |  |  | x |  |  |  |  |  |  |  |  |  |  |  |  |  |
